# Src Reduces Neutrophil Extracellular Traps Generation and Resolves Acute Organ Damage

**DOI:** 10.1002/advs.202506028

**Published:** 2025-08-26

**Authors:** Guotao Lu, Fei Han, Yaodong Wang, Chenchen Yuan, Qingtian Zhu, Tianqi Xia, Lin Chen, Xiaowu Dong, Yanbing Ding, Weiming Xiao, Yuyan Zhang, Jiajia Pan, Hongwei Xu, Weiwei Chen, Bo Tu, Wei Li, Fei Wang, Weijuan Gong, Lianghao Hu

**Affiliations:** ^1^ Pancreatic Center, Department of Gastroenterology The Affiliated Hospital of Yangzhou University Yangzhou University Yangzhou Jiangsu 225000 China; ^2^ Yangzhou Key Laboratory of Pancreatic Disease The Affiliated Hospital of Yangzhou University Yangzhou University Yangzhou Jiangsu 225000 China; ^3^ Department of Gastroenterology Kunshan Hospital of Traditional Chinese Medicine Suzhou Key Laboratory of Integrated Traditional Chinese and Western Medicine of Digestive Diseases Kunshan Affiliated Hospital of Yangzhou University Kunshan 215300 China; ^4^ Department of Intensive Care The Affiliated Hospital of Yangzhou University Yangzhou University Yangzhou 225000 China; ^5^ Medical College Yangzhou University Yangzhou Jiangsu Province 225009 China; ^6^ Clinical Research Division Fred Hutchinson Cancer Research Center Seattle WA 98109 USA; ^7^ Faculty of Pharmaceutical Sciences Toho University Funabashi Chiba 274‐8510 Japan; ^8^ School of Biological Sciences Jinan University Guangzhou 510700 China; ^9^ Department of Gastroenterology Shanghai Institute of Pancreatic Diseases Changhai Hospital Naval Medical University Shanghai 200433 China

**Keywords:** acute pancreatitis, NETs, ROS, sepsis, Src

## Abstract

Neutrophil extracellular traps (NETs) are key factors mediating acute inflammatory injury. However, the underlying mechanisms and potential therapeutic targets remain unclear. Previous results suggest Src may be involved in regulating the NETs formation. Here, Src is found activated in the NETs model in vitro, in the murine‐ and human‐derived neutrophils (acute pancreatitis and sepsis). Moreover, p‐Src expression correlates with the clinical prognosis of acute pancreatitis and sepsis patients. Meanwhile, the inhibition of Src activity (gene silencing or inhibitors) inhibits NETs formation in vitro. Mechanistically, Src directly activates RAF1 by regulating phosphorylation at the Ser 621 site and mediates the RAF/MEK/ERK pathway, thereby affecting the intracellular ROS production. Alternatively, Src activates the RAF/MEK/ERK pathway by mediating PKC phosphorylation. In vivo, neutrophil Src ‐ specific defect significantly reduces acute inflammatory response, organ damage, and the NETs formation in damaged tissue. Eventually, Src inhibitors are used and validated their pharmacological effects. These results identify Src as a key mediator in intracellular ROS production, NETs formation, and acute organ injury. Hence, Src inhibition may represent a promising therapeutic strategy for treating acute organ injury.

## Graphical abstract

Src reduces neutrophil extracellular traps (NETs) generation and resolves acute organ damage. Src directly activates RAF1 by regulating phosphorylation at the Ser 621 site and mediates the RAF/MEK/ERK pathway, thereby affecting the intracellular ROS production. Alternatively, Src activates the RAF1/MEK/ERK pathway by mediating PKC phosphorylation. Src is a key mediator in intracellular ROS production, NETs formation, and acute organ injury. Inhibiting Src may be a promising therapeutic strategy for treating acute organ injury.

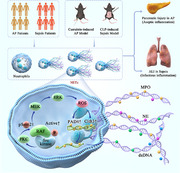

## 1 Introduction

Uncontrolled acute inflammation leads to multiple organ failure, which is one of the main causes of clinical patient mortality and poses a significant challenge to global healthcare. Currently, clinical strategies for treating acute inflammation are limited to systemic steroid therapy, administration of fluids, and antibiotics (only relevant in patients with bacterial infection), but each has its unique limitations.^[^ ^1^ ^]^ Unfortunately, none of the drug experiments in the field of acute inflammatory injury, including sepsis, have shown true benefits.^[^ ^2^ ^]^

Neutrophils are the most abundant innate immune cells in the human body, serving as the central mediator of innate immune responses and playing a central role in acute‐phase reactions.^[^ ^3^ ^]^ During infection or tissue damage, neutrophils take the lead in migrating from circulating blood to the injury site to provide immune protection,^[^ ^4^, ^5^ ^]^ and persistent activation of neutrophils at the site of inflammation is closely related to the severity of the disease.^[^ ^6^ ^]^ The formation of neutrophil extracellular traps (NETs) facilitates the accurate fixation and capture of bacteria, fungi, or viruses by neutrophils, effectively eliminating pathogens.^[^ ^7^ ^]^ However, excessive release and improper resolution of NETs have a significant impact on the pathogenesis of multiple organ damage in acute inflammatory states, including sepsis,^[^ ^8^, ^9^ ^]^ coronavirus disease 2019 (COVID‐19),^[^ ^10^, ^11^ ^]^ acute pancreatitis (AP),^[^ ^12^ ^]^ rheumatic diseases.^[^ ^13^ ^]^ Previous studies had found that NETs formation was associated with the prognosis of various acute inflammatory diseases.^[^ ^14^, ^15^, ^16^ ^]^ Hence, inhibition of NETs release by knocking out peptidylarginine deiminase 4 (PAD4) (one of the key proteins formed by NETs),^[^ ^15^, ^17^ ^]^ directly inhibiting PAD4 via pharmacological inhibitors,^[^ ^18^, ^19^ ^]^ degrading NETs with deoxyribonuclease I (DNase I),^[^ ^20^, ^21^ ^]^ and regulating NETs with the thirteen‐series resolvins (RvTs)^[^ ^22^ ^]^ may prove effective for preventing organ damage in inflammatory diseases related to NETs. Unfortunately, the above intervention methods have not yet been applied in clinical practice.

Proto‐oncogene tyrosine‐protein kinase Src is a tyrosine‐specific kinase that is intimately associated with the pathogenesis of myriad human diseases.^[^ ^23^, ^24^ ^]^ Recent research on Src in granulocytes concentrates mainly on chronic myeloid leukemia.^[^ ^25^ ^]^ In this study, we hypothesize that Src is associated with NETs release related to acute organ injury. Hence, Src pharmacological inhibition may improve the outcome of acute inflammatory diseases.

## 2 Results

### 2.1 Src is Activated in NETs Formation In Vitro and Required for NETs Formation

**Figure 1:**
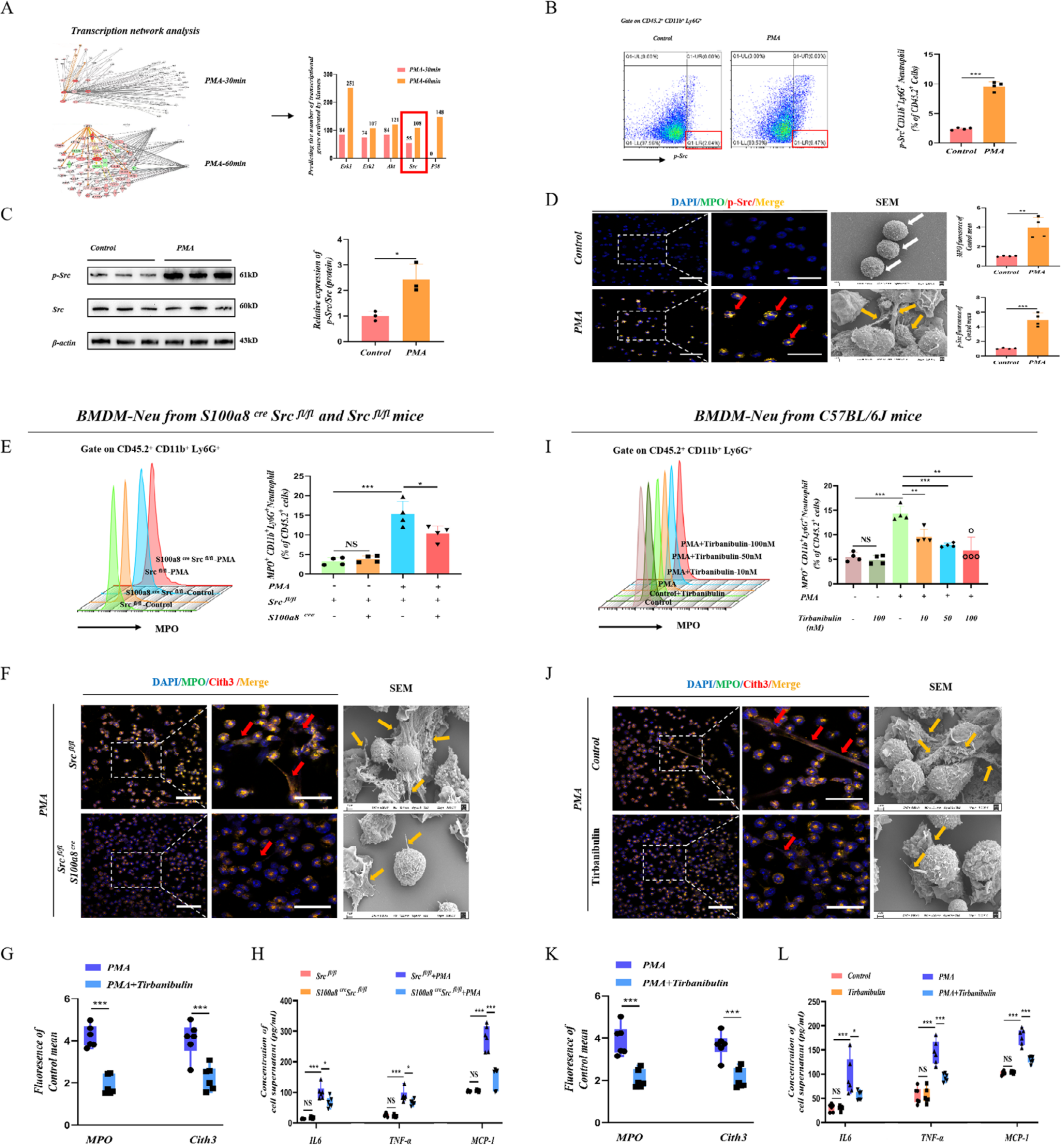
Src kinase is required for the formation of NETs. A) Network analysis at 30‐ and 60‐min for PMA‐mediated NETs formation, predicting the number of transcriptional genes phosphorylated by different kinase cascades activating transcription factors. B) Representative flow cytometry plots and bar graphs depicting the expression levels of p‐Src in neutrophils. Data is presented as mean ± SD (*N* = 4). C) p‐Src protein abundance in neutrophils determined via western blotting (*N* = 3). Relative protein expression of p‐Src (*N* = 3); Src is used as a control for protein loading. D) Representative immunofluorescence image of p‐Src and MPO at 400 × (Scale Bar = 50 µm) and 1000 × (Scale Bar = 10 µm) magnification. Representative SEM of neutrophils between the control and PMA groups. White arrows: neutrophils, yellow arrows: NETs (*N* = 4). Densitometric analysis of p‐Src and MPO fluorescence (*N* = 4). E) Bone marrow neutrophils isolated from S100a8^cre^ Src^fl/fl^ and Src^fl/fl^ mice were cultured and stimulated with PMA (100 nm) for 4 h before flow cytometry analysis. Representative flow cytometry gating of bone marrow neutrophils (CD45.2^+^ CD11b^+^ Ly6G^+^). Representative flow cytometry plots and bar graphs depicting the proportion of neutrophils and the expression level of MPO. Data is presented as mean ± SD (*N* = 4). F) Representative immunofluorescence image of Cith3 and MPO at 400 × (Scale Bar = 50 µm) and 1000 × (Scale Bar = 10 µm) magnification. Representative SEM of neutrophils. White arrows: neutrophils, yellow arrows: NETs (*n* = 6). G) Densitometric analysis of Cith3 and MPO fluorescence (*N* = 6). H) Supernatant levels of IL6, TNF‐α, and MCP‐1 detected using ELISA (*N* = 6). I) Bone marrow neutrophils isolated from C57BL/6J mice cultured and stimulated with PMA (100 nm), and incubated with different doses of tirbanibulin (10, 50, and 100 nm) before flow cytometry analysis. Representative flow cytometry gating of bone marrow neutrophils (CD45.2^+^ CD11b^+^ Ly6G^+^). Representative flow cytometry plots and bar graphs depicting the proportion of neutrophils and the expression level of MPO. Data is presented as mean ± SD (*N* = 4). J) Representative immunofluorescence image of Cith3 and MPO at 400 × (Scale Bar = 50 µm) and 1000 × (Scale Bar = 10 µm) magnification. Representative SEM of neutrophils. White arrows: neutrophils, yellow arrows: NETs (*N* = 6). K) Densitometric analysis of Cith3 and MPO fluorescence (*N* = 6). L) Supernatant levels of IL6, TNF‐α, and MCP‐1 detected using ELISA (*N* = 6). Statistical significance was denoted as: ^*^ *P < 0.05*, ^**^ *P < 0.01*, ^***^ *P < 0.001*.

First, we analyzed open‐source RNA sequencing (RNA‐seq)Kyoto Encyclopedia of Genes and Genomes data to explore new intervention targets for NETs.^[^ ^26^ ^]^ As shown in **Figure** **1**A, the most differentially expressed kinases associated with NETs formation induced by Phorbol‐12‐Myristate‐13‐Acetate (PMA) (a classic inducer of NETs formation) in vitro included extracellular signal‐regulated kinase (ERK)1, ERK2, serine threonine protein kinase (AKT), Src, and P38. Previous studies had shown that Src family kinases and Spleen tyrosine kinase were required for neutrophil extracellular trap formation in response to β‐glucan particles.^[^ ^27^ ^]^ However, the specific function of Src in the formation of NETs requires further investigation. Phosphorylated Src (p‐Src) is the main activation state for the function of Src. Subsequently, flow cytometry, western blotting, and immunofluorescence results revealed that p‐Src was significantly increased in NETs formation in vitro (Figure 1B–D).

To evaluate the correlation between Src and NETs formation in vitro, we generated neutrophil Src‐specific knockout mice (S100a8^cre^ Src^fl/fl^ mice) and assessed the knockout effect of Src in neutrophils (Figure S1A,B, Supporting Information). Results showed that the formation of bone marrow neutrophil NETs in S100a8^cre^ Src^fl/fl^ mice was significantly reduced compared to that in Src^fl/fl^ mice after PMA stimulation (Figure 1E–H; Figure S2A, Supporting Information). Moreover, consistently, the tirbanibulin diminished the generation of NETs induced by PMA in vitro (Figure 1I–L; Figure S2B, Supporting Information). Hence, Src appears to be required for NETs formation.

**Figure 2:**
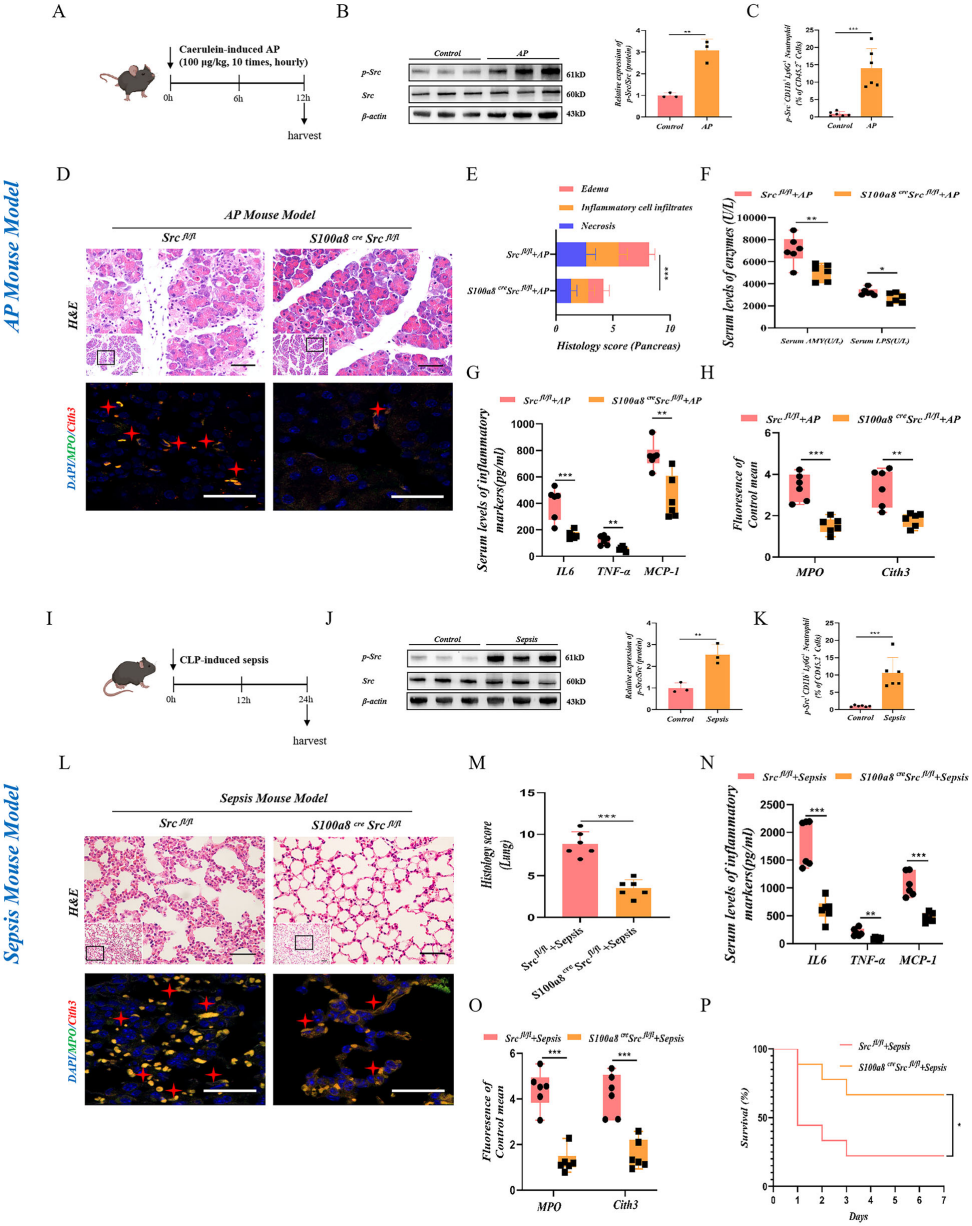
Src‐specific deletion inhibits NETs formation and protects against acute organ damage. A) The AP model is established by intraperitoneal injection of caerulein to S100a8^cre^ Src^fl/fl^ and Src^fl/fl^ mice. B) Protein levels of p‐Src in peripheral blood neutrophils of AP mice were determined using Western blotting (*N* = 3). C) Relative protein expression of p‐Src; Src is used as a control for protein loading (*N* = 3). Representative flow cytometry plots and bar graphs depicting the expression levels of p‐Src. Data is presented as mean ± SD (*N* = 6). D) Representative H&E staining of pancreatic tissues at 100 × (Scale Bar = 100 µm) and 400 × (Scale Bar = 50 µm) magnification. Representative immunofluorescence image of Cith3 and MPO at 1000 × magnification (Scale Bar = 10 µm) (*N* = 6 group^−1^). E) Pathological scores of pancreatic tissues (*N* = 6 group^−1^). F) Serum levels of amylase and lipase (*N* = 6 group^−1^). G) Serum levels of IL6, TNF‐α, and MCP‐1 detected by ELISA (*N* = 6 group^−1^). H) Densitometric analysis of Cith3 and MPO fluorescence (*N* = 6 group^−1^). I) Sepsis model is established by CLP to S100a8^cre^ Src^fl/fl^ and Src^fl/fl^ mice. J) Protein levels of p‐Src in peripheral blood neutrophils of sepsis mice were determined using Western blotting (*N* = 3). Relative protein expression of p‐Src; Src is used as a control for protein loading (*N* = 3). K) Representative flow cytometry plots and bar graphs depicting the expression levels of p‐Src. Data is presented as mean ± SD (*N* = 6). L) Representative H&E staining of lung tissues at 100 × (Scale Bar = 100 µm) and 400 × (Scale Bar = 50 µm) magnification. Representative immunofluorescence image of Cith3 and MPO at 1000 × magnification (Scale Bar = 10 µm; *N* = 6 group^−1^). M) Pathological scores of lung tissues (*N* = 6 group^−1^). *N*) Serum levels of IL6, TNF‐α, and MCP‐1 detected by ELISA (*N* = 6 group^−1^). O) Densitometric analysis of Cith3 and MPO fluorescence (*N* = 6 group^−1^). P) Survivorship curve of mice in sepsis model induced by CLP (*N* = 10 group^−1^). Statistical significance was denoted as: ^*^ *P* < 0.05, ^**^ *P* < 0.01, ^***^ *P* < 0.001.

### 2.2 Src‐Specific Deletion Inhibits NETs Formation and Protects against Acute Organ Damage in Mice

Subsequently, we conducted in vivo experiments. As mentioned above, two classic animal models of acute inflammatory injury were used to validate the function of Src in vivo. A typical AP injury mouse model was induced by cerulein, characterized by significant edema, infiltration of inflammatory cells, necrosis of pancreatic acinar cells, and significant elevation of serum amylase, lipase, and inflammatory factor levels (**Figure** **2**A). Following the isolation of neutrophils from the peripheral blood of mice with AP, western blotting and flow cytometry revealed increased levels of p‐Src levels in circulating neutrophils under acute inflammatory injury compared to those in the normal control group (Figure 2B,C). Compared with the control group (Src^fl/fl^ mice), neutrophil Src‐specific deficient mice (S100a8^cre^ Src^fl/fl^ mice) showed significantly reduced pancreatic tissue pathological damage. Moreover, compared with the neutrophil Src‐specific deficient mice (S100a8^cre^ Src^fl/fl^ mice), the control group (Src^fl/fl^ mice) showed serum amylase, lipase, and inflammatory factor levels were markedly increased, accompanied by obviously increased NETs formation in pancreatic tissue (Figure 2D–H).

As shown in Figure 2I, we constructed classic CLP‐induced sepsis mouse models to infectious inflammatory injuries. Following the isolation of neutrophils from the peripheral blood of mice with AP, western blotting and flow cytometry revealed increased levels of p‐Src levels in circulating neutrophils under acute inflammatory injury compared to those in the normal control group (Figure 2J,K). Src^fl/fl^ mice exhibited the marked acute lung injury phenotype, mainly characterized by pulmonary vascular congestion, edema, significant enhancement of neutrophil infiltration, and elevated serum inflammatory factor levels, while the aforementioned pathological lesions were significantly reduced in S100a8^cre^ Src^fl/fl^ mice (Figure 2L–N). We also observed a marked reduction in NETs formation in the lung tissue of S100a8^cre^ Src^fl/fl^ mice compared with Src^fl/fl^ mice (Figure 2L,O). In fact, in the context of sepsis induced by CLP, the survival rate of S100a8^cre^ Src^fl/fl^ mice was also improved compared to control mice (Figure 2P).

To sum up, these in vivo results confirm that neutrophil Src was closely related to organ damage and NETs formation in acute inflammatory diseases.

### 2.3 Src Promotes Intracellular ROS Production through the RAF/MEK/ERK Signaling Pathway

**Figure 3:**
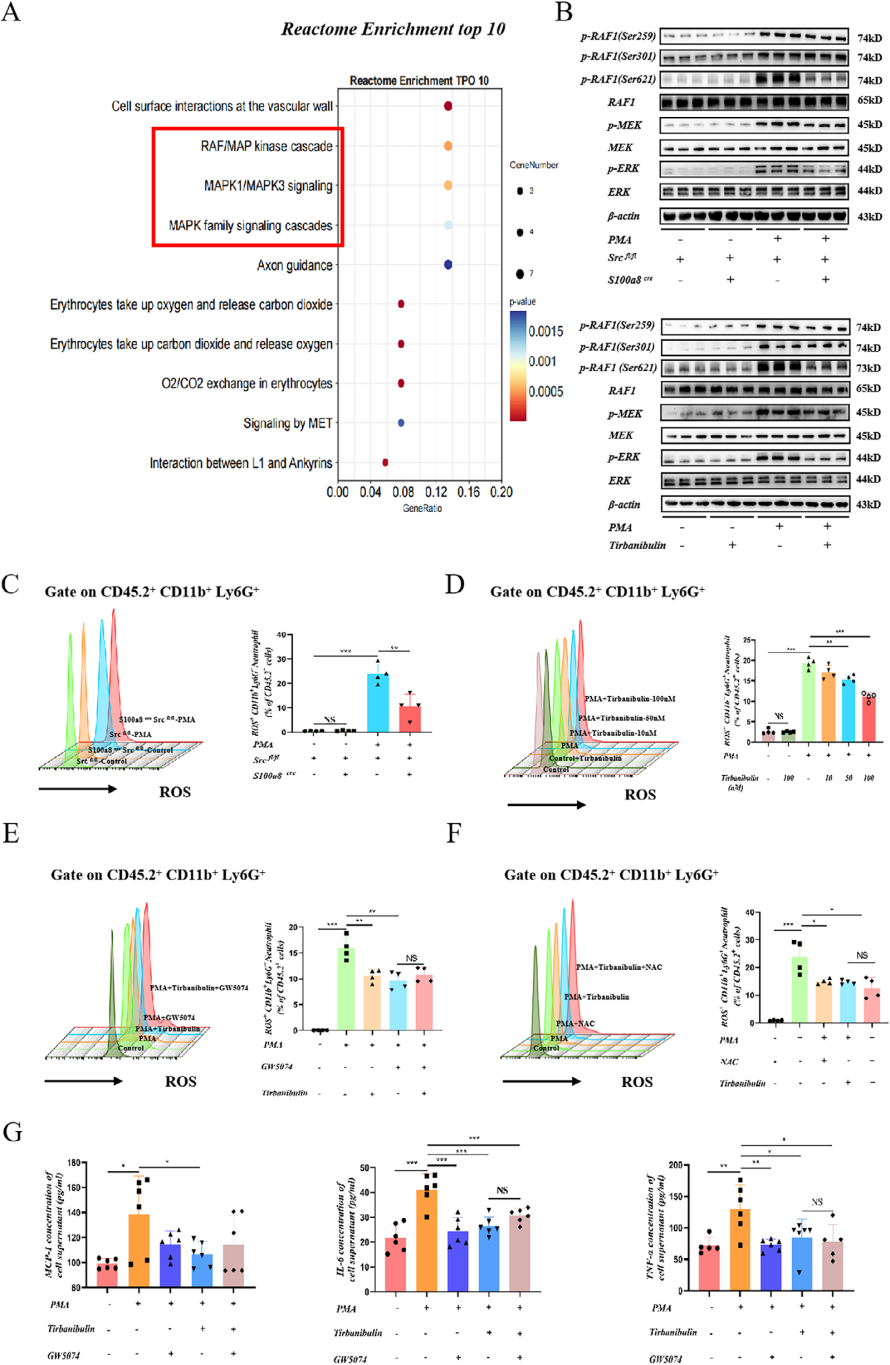
Src regulates intracellular ROS production through the RAF/MEK/ERK signaling pathway. A) Reactome enrichment analysis of the differential genes of S100a8^cre^ Src^fl/fl^ + PMA and Src^fl/fl^ + PMA mice (*N* = 3). B) The protein levels of p‐RAF1, p‐MEK, and p‐ERK in neutrophils of Src^fl/fl^, S100a8^cre^ Src^fl/fl^, PMA+Src^fl/fl^, and PMA+S100a8^cre^ Src^fl/fl^ groups determined by western blotting (*N* = 3). The protein levels of p‐RAF1, p‐MEK, and p‐ERK in neutrophils of four groups (Control, Control + Tirbanibulin, PMA, PMA + Tirbanibulin) assessed using western blotting (*N* = 3). C) Bone marrow neutrophils isolated from S100a8^cre^ Src^fl/fl^ and Src^fl/fl^ mice cultured and stimulated with PMA (100 nm) for 4 h before flow cytometry analysis. Representative bar graphs depicting the proportion of neutrophils and the expression level of ROS. Data is presented as mean ± SD (*N* = 4). D) Bone marrow neutrophils isolated from C57BL/6J mice cultured and stimulated with PMA (100 nm), and incubated with different doses of tirbanibulin (10, 50, and 100 nm) before flow cytometry analysis. Representative bar graphs depicting the proportion of neutrophils and the expression level of ROS. Data is presented as mean ± SD (*N* = 4). E) Representative bar charts of ROS expression level in five groups: Control, PMA, PMA+Tirbanibulin, PMA+GW5074, and PMA+Tirbanibulin+GW5074. Data is presented as mean ± SD (*N* = 4). F) Representative bar charts of ROS expression level in five groups: Control, PMA, PMA+Tirbanibulin, PMA+NAC, and PMA+Tirbanibulin+NAC. Data is presented as mean ± SD (*N* = 4). G) Supernatant levels of IL‐6, TNF‐α, and MCP‐1 detected by ELISA (*N* = 6). Statistical significance was denoted as: ^*^ *P < 0.05*, ^**^ *P < 0.01*, ^***^ *P < 0.001*.

To elucidate the molecular mechanism associated with the role of Src in NETs formation, we performed RNA‐seq on RNA from the bone marrow neutrophils of S100a8^cre^ Src^fl/fl^ mice and Src^fl/fl^ mice induced by PMA in vitro. Reactome enrichment analysis revealed significant changes in the Rapidly Accelerated Fibrosarcoma/Mitogen‐Activated Protein Kinase (RAF/MAPK) signaling pathway, which is a classical signal pathway for mediating the production of reactive oxygen species (ROS) and promoting the formation of NETs^[^ ^25^ ^]^ (**Figure** **3**A). To improve the reliability of our enriched pathway, we first predicted the targets of Src kinase inhibitors (Tirbanibulin, Bosutinib, Dasatinib) using the SwissTarget Prediction website. Then, we combined the predicted targets of Src kinase inhibitors with our RNA‐seq data (RNA‐seq analysis of PMA‐induced S100a8^cre^ Src^fl/fl^ mice and Src^fl/fl^ mice bone marrow neutrophils). Kyoto Encyclopedia of Genes and Genomes (KEGG) enriched pathway analysis revealed significant changes in the MAPK signaling pathway (Figure S3, Supporting Information). This is consistent with the results of the Reactome enrichment analysis above.

Subsequent Polymerase Chain Reaction (PCR) and western blotting analysis revealed that the protein abundances of p‐RAF1(Ser 621), p‐MEK, and p‐ERK were significantly increased after PMA stimulation compared to the control group, and significantly decreased after inhibiting Src activity (gene mutation or inhibitors) (Figure 3B; Figures S4 and S5, Supporting Information). Clearly, targeting inhibition of Src activity (pharmacological inhibition or gene knockout) significantly reduced the production of ROS in the formation of NETs in vitro (Figure 3C,D). Consistent with previous reports, RAF inhibitor (GW5074) markedly reduced NETs formation in vitro (Figure S6, Supporting Information). On the basis of the GW5074 intervention, the combination therapy of tirbanibulin and GW5074 did not further inhibit the formation of NETs (Figure 3E; Figure S7, Supporting Information), which was verified by the data of inflammatory factors in cell supernatant (Figure 3G). ROS was crucial in the formation of NETs, and NAC (a classic ROS scavenger) significantly inhibited the NETs formation (Figure S8, Supporting Information). Furthermore, on the basis of NAC intervention, we confirmed that the combined treatment of tirbanibulin and NAC did not further inhibit the formation of the NETs (Figure 3F; Figure S9, Supporting Information). Collectively, these results suggested that Src may promote PMA‐induced NETs formation by mediating ROS production through the RAF/MEK/ERK signaling pathway.

**Figure 4:**
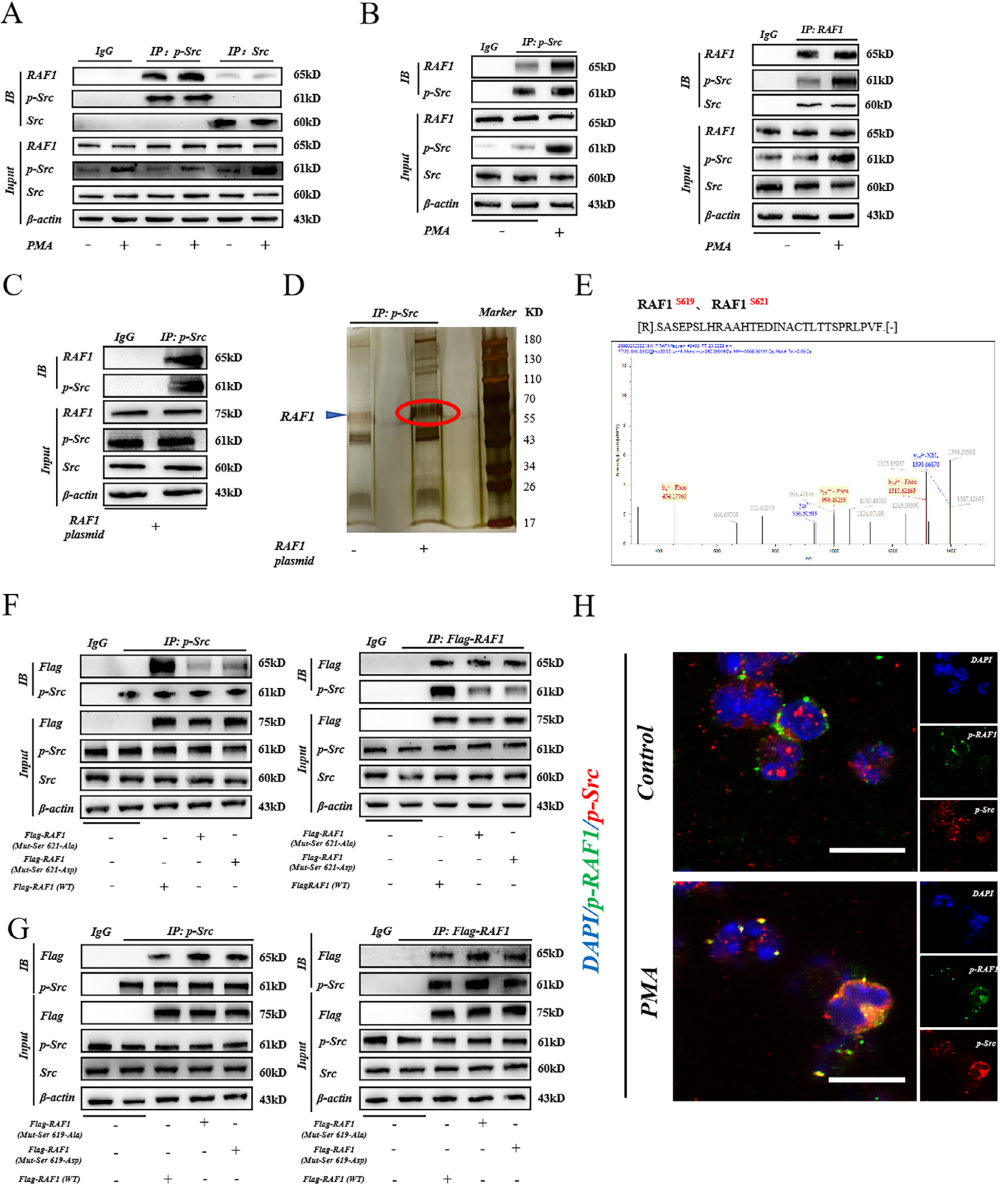
p‐Src phosphorylates RAF1 at the Ser 621 site. A) Co‐IP of the interaction between Src and p‐Src with RAF1 in C57BL/6J mouse bone marrow neutrophils before and after PMA stimulation. B) Co‐IP of the interaction between p‐Src and RAF1. Neutrophil lysates isolated from the bone marrow of C57BL/6J mice assessed by Co‐IP with anti‐Src Family (photo Y418) antibody or anti‐RAF1 antibody. C) Co‐IP of the interaction between p‐Src and RAF1 in HEK‐293T cells. D) SDS‐PAGE separation and silver staining of protein Co‐IP with anti‐Src Family (photo Y418) antibody. E) Mass spectrometric analysis identified phosphorylated RAF1 at S619 and S621. Secondary spectra for the phosphorylated peptides are shown. F) Immunoprecipitation analysis of the interaction between anti‐Src Family (photo Y418) antibody and RAF1 in HEK‐293T cells transfected with RAF1 (WT, Mut‐Ser 621‐Ala, Mut‐Ser 621‐Asp) plasmids. Immunoprecipitation analysis of interaction between anti‐Flag immunomagnetic beads and p‐Src in HEK‐293T cells transfected with RAF1 (WT, Mut‐Ser 621‐Ala, Mut‐Ser 621‐Asp) plasmids. G) Immunoprecipitation analysis of the interaction between anti‐Src Family (photo Y418) antibody and RAF1 in HEK‐293T cells transfected with RAF1 (WT, Mut‐Ser 619‐Ala, Mut‐Ser 619‐Asp) plasmids. Immunoprecipitation analysis of interaction between anti‐Flag immunomagnetic beads and p‐Src in HEK‐293T cells transfected with RAF1 (WT, Mut‐Ser 619‐Ala, Mut‐Ser 619‐Asp) plasmids. H) Representative immunofluorescence image of p‐Src and p‐RAF1 at 2500 × (Scale Bar = 5 µm) magnification.

Subsequently, we investigated the specific molecular biology mechanisms. The direct interaction was observed between p‐Src or Src and RAF1 via Co‐Immunoprecipitation (Co‐IP), and the interaction effect between p‐Src and RAF1 was more significant than that of Src (**Figure** **4**A,B). Considering that RAF1 typically contains multiple phosphorylated active sites, we sought to determine which specific site p‐Src interacts with to activate RAF1. To this end, we constructed the RAF1 overexpression plasmid, collected and lysed HEK‐293T cells transfected with Flag‐RAF1, and used an anti‐p‐Src antibody for Co‐IP and silver staining analysis (Figure 4C,D). The results showed that the p‐Src phosphorylated RAF1 at Ser 619 and Ser 621 (Figure 4E).

Typically, two types of mutations affect phosphorylation: 1) substitution to aspartic acid (D), which mimics continuous phosphorylation, and 2) substitution to alanine (A) which inhibits the phosphorylation ability of the kinase. Co‐IP analysis using an anti‐p‐Src antibody showed that when RAF1 Ser 621 was mutated, the interaction between p‐Src and RAF1 was significantly inhibited compared to that in the control group; however, this effect was not observed following the RAF1 Ser 619 mutation (Figure 4F,G). Consistent results were obtained for Co‐IP using FLAG immunomagnetic beads. Immunofluorescence staining also confirmed this point (Figure 4H). Hence, p‐Src activates RAF1 by regulating phosphorylation at the Ser 621 site. Interestingly, the Ser 621 site of RAF1 was the same as the previous western blotting results (Figure 3B).

It was widely known that protein kinase C (PKC) was a recognized mediator in PMA‐induced NETs formation. Previous studies had shown that PMA stimulation activated NADPH oxidase in neutrophils through the PKC and Raf‐MEK‐ERK signaling pathways, leading to the production of ROS, which was one of the key mechanisms for the formation of NETs.^[^ ^28^, ^29^ ^]^ Therefore, we further investigated the protein interactions between p‐Src and PKC. As shown in Figure S10 (Supporting Information), the Co‐IP experiment revealed the direct interaction between p‐Src and PKC. Previous studies had shown that PKC inhibitors reduced the proinflammatory and tissue damage effects of neutrophils in COVID‐19.^[^ ^30^ ^]^ The deficiency of Geranylgeranyl diphosphate synthase in neutrophils could promote the NETs formation through PKC/NOX signaling, thereby exacerbating lipopolysaccharide (LPS) induced lung injury.^[^ ^31^ ^]^ In Figure S11 (Supporting Information), we found through the classical NETs model induced by PMA in vitro that inhibiting PKC significantly reduced the NETs formation. Subsequently, as shown in Figure S12 (Supporting Information), compared with the control group, the protein abundance of p‐PKC significantly increased after PMA stimulation, and markedly decreased after inhibition of Src activity (gene mutation or inhibitor).

PMA, a classical PKC agonist, activated PKC by directly binding to its C1 domain, stabilizing the active conformation and thereby inducing membrane translocation.^[^ ^32^ ^]^ To better elucidate the alignment between the established mechanisms of p‐Src‐mediated PKC activation and PMA‐induced PKC activation, we further validated this using the LPS‐induced NETs model. As shown in Figure S13 (Supporting Information), Co‐IP assays revealed a direct interaction between p‐Src and PKC, consistent with observations in the PMA‐induced NETs model. From this, in both PMA and LPS induced NETs models, p‐Src bound to and activated PKC, establishing p‐Src‐mediated PKC activation as an integral component of the signaling pathway. Therefore, p‐Src‐mediated and PMA‐induced PKC activation might function cooperatively.

To sum up, on the one hand, Src activated RAF1 by regulating phosphorylation at the Ser 621 site and mediated the RAF/MEK/ERK pathway, thereby affecting the intracellular ROS production. On the other hand, Src also mediated PKC phosphorylation, which subsequently activated the RAF1/MEK/ERK pathway, thereby affecting NETs formation. The two pathways together strengthened the crucial role of Src in NETs formation.

### 2.4 Pharmacological Inhibition of Src Inhibits the Release of NETs and Acute Organ Damage in Mice

**Figure 5:**
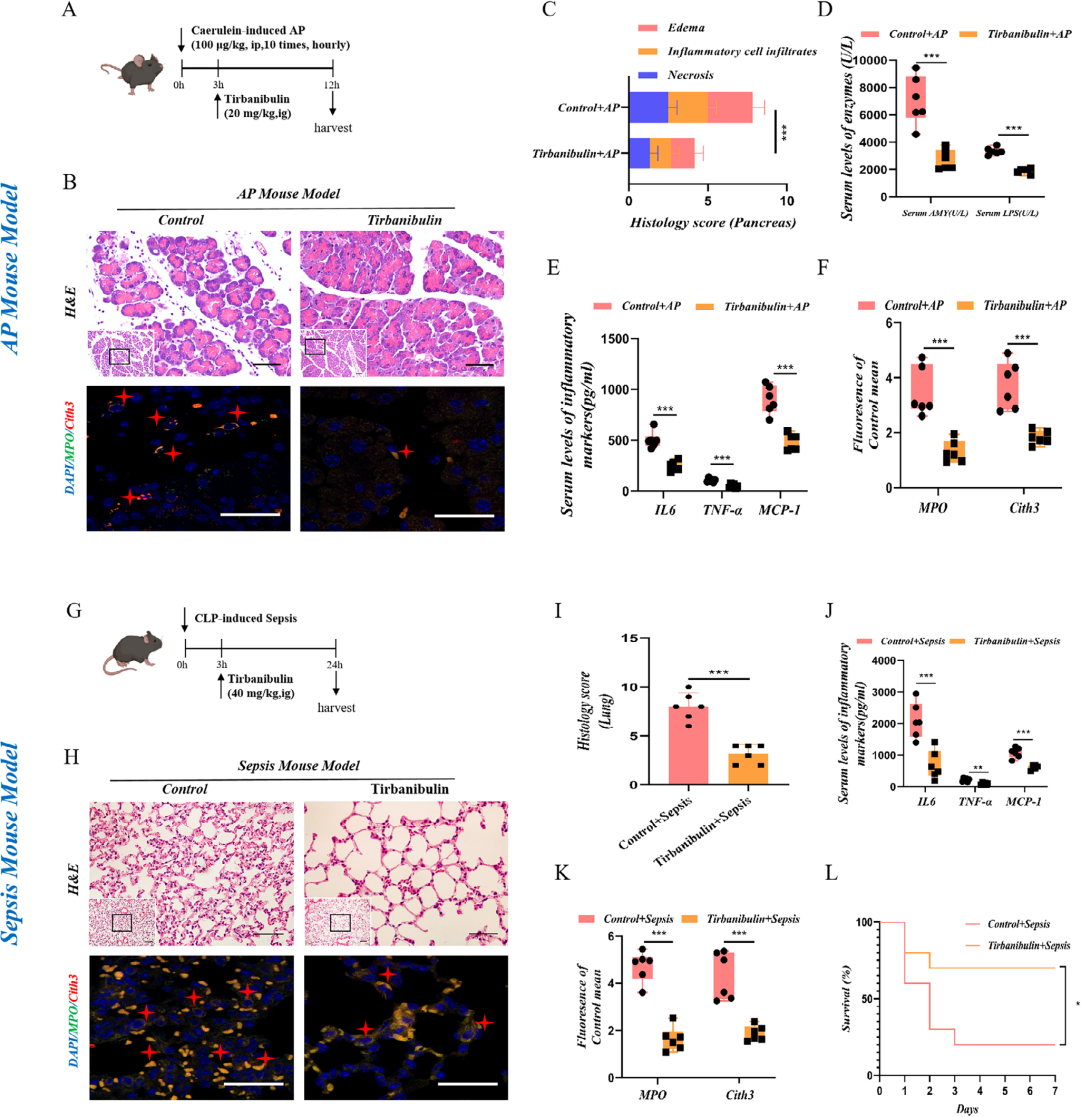
Tirbanibulin represses the release of NETs and acute organ damage in AP and sepsis murine models. A) The AP model is established via intraperitoneal injection of caerulein to C57BL/6J mice. Three hours after the first injection, mice are gavaged with tirbanibulin (20 mg kg^−1^). B) Representative H&E staining of pancreatic tissues at 100 × (Scale Bar = 100 µm) and 400 × (Scale Bar = 50 µm) magnification. Representative immunofluorescence image of Cith3 and MPO at 1000 × magnification (Scale Bar = 10 µm; *N* = 6 group^−1^). C) Pathological scores of pancreatic tissues (*N* = 6 group^−1^). D) Serum levels of amylase and lipase (*N *= 6 group^−1^). E) Serum levels of IL‐6, TNF‐α, and MCP‐1 detected by ELISA (N = 6 group^−1^). F) Densitometric analysis of Cith3 and MPO fluorescence (*N* = 6 group^−1^). G) The sepsis model is established by CLP in C57BL/6J mice. Three hours after surgery, mice were given tirbanibulin (40 mg kg^−1^) by gavage. H) Representative H&E staining of lung tissues at 100 × (Scale Bar = 100 µm) and 400 × (Scale Bar = 50 µm) magnification. Representative immunofluorescence image of Cith3 and MPO at 1000 × magnification (Scale Bar = 10 µm; *N* = 6 group^−1^). I) Pathological scores of lung tissues (*N* = 6 group^−1^). J) Serum levels of IL‐6, TNF‐α, and MCP‐1 detected by ELISA (*N* = 6 group^−1^). K) Densitometric analysis of Cith3 and MPO fluorescence (*N* = 6 group^−1^). L) Survivorship curve of mice in sepsis model induced by CLP (*N* = 9 group^−1^). Statistical significance was denoted as: ^*^ *P < 0.05*, ^**^ *P < 0.01*, ^***^ *P < 0.001*.

Subsequently, we conducted in vivo experiments following the treatment of both murine disease models with Src inhibitors‐ tirbanibulin, and two non‐specific inhibitors (bosutinib and dasatinib)‐to observe their pharmacological effects. As shown in the experimental flowchart, we focused on the therapeutic effect of drugs on disease models (**Figure** **5**A,G). Histopathological damage to pancreatic tissue, NETs formation in pancreatic tissue, serum amylase, lipase, and inflammatory factor levels were significantly reduced in AP mice treated with tirbanibulin compared to those in the control group (Figure 5B–F; Figure S14, Supporting Information). Similarly, lung histological damage was also obviously mitigated in sepsis mice treated with tirbanibulin; the immunofluorescence results and serum inflammatory factor levels were consistent with those of the AP model (Figure 5H–K; Figure S15, Supporting Information). Additionally, an improvement in the overall survival rate was observed following tirbanibulin treatment (Figure 5L). Similarly, as expected, the non‐specific Src inhibitors (bosutinib and dasatinib) also exhibited positive therapeutic efficacy in both models (Figures S16 and S17, Supporting Information). Hence, drug‐induced inhibition of Src activity reduced NETs release in acute inflammatory diseases and alleviated acute organ dysfunction.

### 2.5 p‐Src Abundance is Significantly Increased in Human Peripheral Blood Neutrophils and is Closely Associated with Disease Prognosis

Eventually, we assessed the abundance of p‐Src in the peripheral blood neutrophils of clinical patients. Corresponding to the mouse models, we included AP and sepsis patients, and the clinical characteristics of patients were shown in Tables S1 and S2 (Supporting Information). Based on the presence of local pancreatic complications (LC), we divided patients with AP into a non‐LC group (*N* = 41) and an LC group (*N* = 33). Meanwhile, patients with sepsis were divided into survivors (*N* = 23) and non‐survivors (*N* = 17) groups.

**Figure 6:**
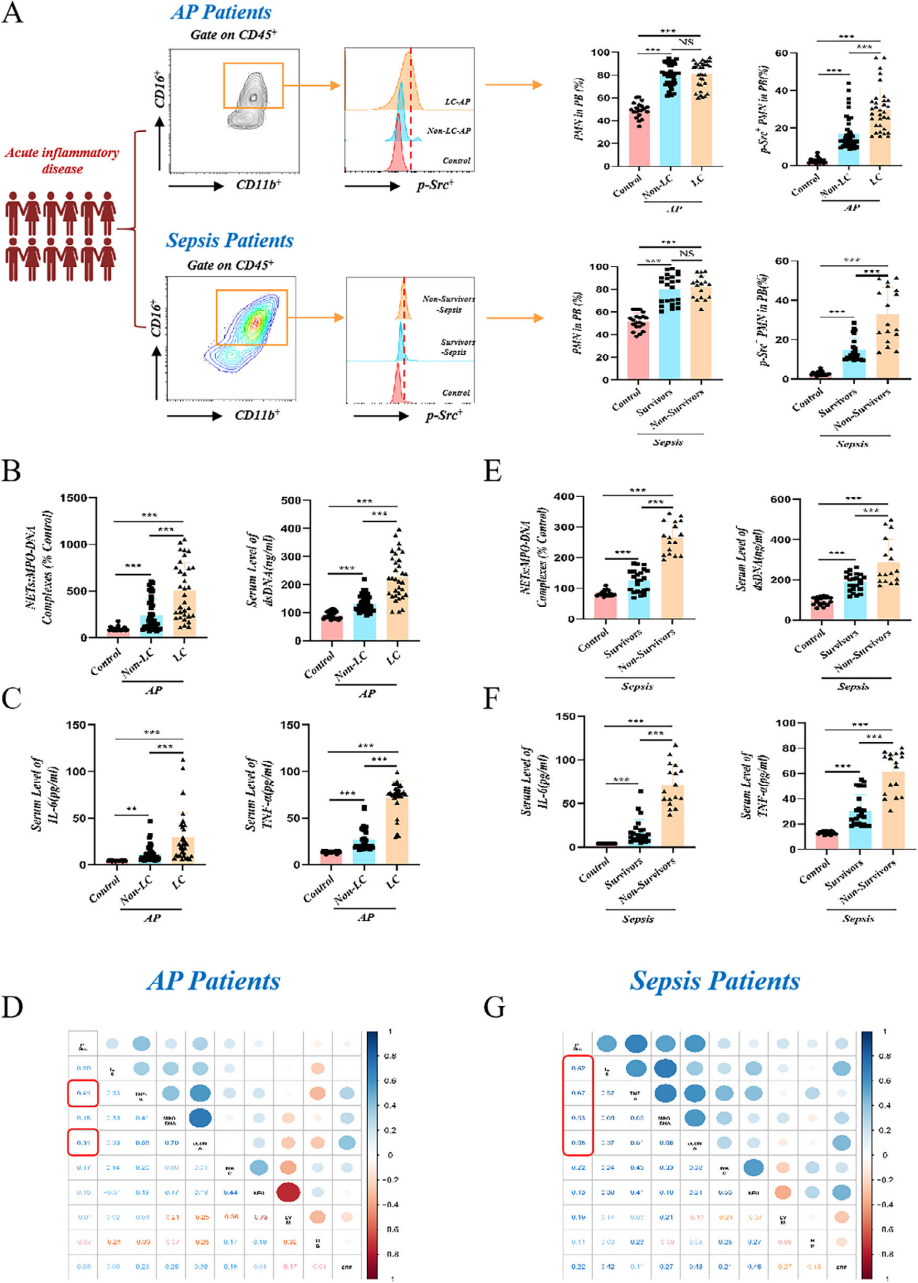
Phosphorylation of Src is significantly increased in peripheral blood neutrophils and is associated with disease prognosis. A) Representative flow cytometry gating of human peripheral blood neutrophil cells (CD45^+^ CD11b^+^ CD16^+^). Representative flow cytometry plots and bar graphs depicted the expression levels of p‐Src in neutrophils. Data is presented as mean ± SD. Neutrophils isolated from the peripheral blood of controls (*N* = 20), AP patients with non‐local complications (Non‐LC AP, *N* = 41), AP patients with local complications (LC AP, *N* = 33), survivors sepsis patients (*N* = 23), and Non‐survivors sepsis patients (*N* = 17). B) Plasma NETs levels (MPO‐DNA Complexes and dsDNA) across all groups: control (*N* = 20), non‐LC AP patients (*N* = 41), LC AP patients (*N* = 33). The *y*‐axis depicts plasma MPO‐DNA complexes expressed as a percentage of controls ± SD, arbitrarily set at 100%. C) Plasma IL‐6 and TNF‐α levels across all groups: control (*N* = 20), non‐LC AP patients (*N* = 41), LC AP patients (*N* = 33). D) Heatmap of correlation coefficients between serum p‐Src levels and other clinical indices in AP patients. E) Plasma NETs levels (MPO‐DNA complexes and dsDNA) across all groups: control (*N* = 20), survivors sepsis patients (*N* = 23), Non‐survivors sepsis patients (*N* = 17). The *y*‐axis depicts plasma MPO‐DNA complexes expressed as a percentage of controls ± SD, arbitrarily set at 100%. F) Plasma IL‐6 and TNF‐α levels across all groups: control (*N* = 20), survivors sepsis patients (*N* = 23), Non‐survivors sepsis patients (*N* = 17). G) Heatmap of correlation coefficients between serum p‐Src levels and other clinical indices in sepsis patients. Statistical significance was denoted as: ^**^ *P < 0.01*, ^***^ *P < 0.001*.

In patients with non‐infectious (AP; **Figure** **6**A–C) or infectious (sepsis; Figures 2, 6) acute inflammatory injury, the expression of p‐Src in peripheral blood neutrophils, and serum NETs‐related indicators (MPO‐DNA complex and dsDNA), and pro‐inflammatory factors (IL‐6 and TNF‐α) were markedly increased compared to the healthy control group; these indicators were positively associated with poor prognosis. In addition, variable correlation heatmap analysis and Pearson correlation analysis identified positive correlations between circulating neutrophil p‐Src levels and serum pro‐inflammatory factors (TNF‐α, etc.) and NETs‐related indicators (dsDNA, etc.) (Figure 6D,G; Figure S18, Supporting Information).

These results indicated that circulating neutrophils in patients with acute inflammatory injury express increased levels of p‐Src, which correlates with disease prognosis.

## 3 Discussion

Our research confirms the crucial role of Src in intracellular ROS generation and NETs formation. In particular, we employed classic AP and sepsis mouse models to demonstrate that Src inhibition via gene knockout or pharmacological treatment could prevent NETs release, reduce acute inflammatory organ damage, and improve disease prognosis. Importantly, we also showed that the p‐Src levels in the neutrophils of patients with acute inflammatory diseases (AP and sepsis) were significantly elevated and closely associated with disease prognosis.

Src is widely expressed in myriad cell types and functions as a key regulatory factor in cell signal transduction, playing a crucial role in pathological and physiological processes, such as cell development and tumor formation.^[^ ^33^, ^34^ ^]^ Previous research on Src focused primarily on malignant tumors, including chronic myeloid leukemia and breast tumors.^[^ ^25^, ^35^ ^]^ However, recently, its potential to regulate the immune system has been preliminarily explored, revealing that Src mediates multiple signaling pathways involved in inflammatory disease damage.^[^ ^36^, ^37^ ^]^ Kao et al. found that inhibiting the phosphorylation of the Src family could regulate neutrophil inflammation.^[^ ^36^ ^]^ Meanwhile, in a rat model of osteoarthritis, Src activates the signal transducer and activators of transcription 3 (STAT3)/activated mitogen‐activated protein kinase (MAPK) pathway, exacerbating the tissue damage.^[^ ^36^ ^]^ Through in vitro and in vivo experiments and clinical data analysis, we verified that Src was involved in neutrophil‐mediated innate immune responses, further confirming the regulatory effects of Src on inflammatory immunity.

The excessive formation of NETs is closely related to the production of excessive ROS in neutrophils, which are key mediators of cellular oxidative stress. The RAF/MEK/ERK signaling pathway is a key pathway that promotes the generation of ROS and is also an important upstream factor in triggering the release of NETs.^[^ ^38^, ^39^ ^]^ Meanwhile, RAF1 is a key kinase associated with NETs formation that activates nicotinamide adenine dinucleotide phosphate hydride (NADPH), mediates the production of large amounts of ROS by neutrophils, and promotes the release of NETs.^[^ ^40^ ^]^ In the current study, RNA‐seq and subsequent functional studies revealed that inhibiting Src might block NETs formation by regulating the RAF/MEK/ERK signaling pathway. Moreover, we found that Src regulated RAF1 phosphorylation at Ser 621, which was conducive to reduce ROS generation and subsequent release of NETs. This emphasized the important interaction between ROS, Src activity, and NETs formation. Nevertheless, Src might also interact with other signaling pathways.

PKC was a recognized mediator in PMA‐induced NETs formation. PMA stimulation activated NADPH oxidase in neutrophils through the PKC and RAF/MEK/ERK signaling pathways, leading to the production of ROS, which was one of the key mechanisms for the formation of NETs.^[^ ^28^, ^29^ ^]^ We found that direct interaction between p‐Src and PKC, and inhibiting PKC significantly reduced the PMA‐induced NETs. Besides that, previous research has shown that PMA activates PKC by directly binding to its C1 domain, stabilizing the active conformation and thereby inducing membrane translocation.^[^ ^32^ ^]^ To investigate the consistency between the established mechanisms of Src‐mediated PKC activation and PMA‐induced PKC activation, we validated two NETs models (LPS and PMA induction). It was found that in both PMA and LPS‐induced NETs models, Src bound to and activated PKC. From this, Src also activates the RAF/MEK/ERK pathways through PKC phosphorylation, promoting ROS production and subsequent NETs release. In summary, Src phosphorylates RAF1 at Ser‐621, triggering the RAF/MEK/ERK cascade and modulating intracellular ROS production. Simultaneously, Src induces PKC phosphorylation, which subsequently activates the RAF1/MEK/ERK pathway to drive NETs formation. However, Src may also interact with other signaling pathways, which deserves further investigation.

In infections and aseptic diseases, NETs directly killed epithelial and endothelial cells, while the excessive release of NETs damages the normal physiological function of tissues, even leading to organ failure.^[^ ^38^ ^]^ However, currently, there are no effective interventions that could inhibit NETs release in clinical practice. Src inhibitor (tirbanibulin) and non‐specific inhibitors (bosutinib and dasatinib) are orally active inhibitors that suppress Src activity. Herein, we demonstrated the pharmacological targeting effect of Src in NETs formation, indicating that Src inhibitors have the potential for clinical application in acute inflammatory diseases. Crucially, in this study, besides the inhibitor (tirbanibulin), we also evaluated the effects of two non‐specific inhibitors (bosutinib and dasatinib) commonly used in clinical practice with good conversion potential. However, this requires the evaluation of their clinical efficacy in treating patients with acute inflammatory injury in subsequent clinical trials.

Overall, our results indicated that Src was active in circulating neutrophils during acute inflammatory diseases and played a crucial role in NETs release from damaged tissues and organs. Importantly, Src inhibitors improved acute inflammatory diseases and subsequent organ damage and had potential clinical applications.

## 4 Experimental Section

### Materials Availability

This study did not generate new reagents.

### Participant and Blood Sample Collection

Patients were recruited with AP and sepsis admitted to the Yangzhou University Affiliated Hospital between July 2021 and May 2023. AP was diagnosed according to the revised Atlanta diagnostic criteria in 2012;^[^ ^41^ ^]^ 74 AP cases were included and their demographic, clinical, and laboratory parameters were collected. The exclusion criteria were as follows: 1) <18 or >80 years old, 2) pregnant, 3) previous history of malignant tumors, 4) chronic or recurrent pancreatitis, and 5) no systematic laboratory evaluation or incomplete information. Patients with systemic or local infections were excluded. Sepsis was diagnosed according to the Second International Sepsis Definition Standard;^[^ ^42^ ^]^ 40 sepsis cases were included and demographic, clinical, and their laboratory parameters were collected. The exclusion criteria were as follows: 1) active hematological malignancies, 2) pregnancy, 3) long‐term hormone therapy, 4) transplantation, 5) human immunodeficiency virus infection, 6) advanced liver cirrhosis, 7) lack of systematic laboratory evaluation, and 8) incomplete information. Peripheral blood samples were collected from the patients during the acute phase of the disease. Besides that, twenty healthy volunteers were recruited as controls for AP and sepsis. All participants in this study obtained informed written consent from all participants or close relatives. The study was approved by the Ethics Committee of the Yangzhou University Affiliated Hospital (No. 2018‐YKL11‐27‐(Topic 3)).

### Animals

C57BL/6J mice (20–25 g), S100a8^cre^ mice, and Src^floxp^ mice were purchased from GemPharmatech Co., Ltd., Nanjing, China. S100a8^cre^ mice were bred with Src^fl/fl^ mice to produce Src‐specific neutrophil knockout (S100a8^cre^ Src^fl/fl^) offspring. All animals were housed under specific pathogen‐free conditions, with a 12 h light/dark cycle and a temperature range of 21–25 °C. All animal experiments were conducted by the Principles of Laboratory Animal Care (NIH publication No. 85Y50, revised 1996) and the ARRIVE guidelines, and approved by the Experimental Animal Ethics Committee of Yangzhou University (No. 202 208 012).

### Animal Models and Sample Collection

The classical AP mouse model was created using cerulein according to a previously published protocol.^[^ ^43^ ^]^ In brief, mice were fasted overnight, and AP was induced via intraperitoneal injection of caerulein (100 µg kg^−1^, 1 h intervals, 10 times), while the control mice received the same volume of phosphate‐buffered saline (PBS). All mice were anesthetized and euthanized after 12 h.

The sepsis mouse model was generated via cecal ligation and puncture (CLP) according to a previously described protocol.^[^ ^44^ ^]^ In simpler terms, mice were temporarily anesthetized and performed two punctures through the cecum using an 18‐gauge needle to induce severe sepsis. Control mice (sham‐operated group) underwent the same surgery without cecal puncture. All mice received a subcutaneous injection of 1 mL of physiological saline immediately after surgery. All mice were anesthetized and euthanized after 24 h. Blood samples were collected 24 h after surgery, and the mice were euthanized under anesthesia. Additionally, the survival period was up to 7 days.

Blood samples were collected from the inner canthus of mice and processed to evaluate serum amylase, lipase, and cytokine levels. Moreover, following euthanasia, pancreatic and lung tissue samples were collected. A portion of the tissues was fixed in 4% paraformaldehyde and embedded in paraffin blocks for hematoxylin and eosin staining. The remaining pancreatic and lung tissues were frozen at −80 °C for later use.

### Src Inhibitors Treatment

Three hours after modeling AP and sepsis, mice were treated with the control vector (0.5% CMC‐Na solution) or Src inhibitors (tirbanibulin, bosutinib, or dasatinib) by gavage. In the AP mouse model, the administration group mice were given tirbanibulin (5, 10, and 20 mg kg^−1^) or bosutinib (20, 40, and 80 mg kg^−1^) or dasatinib (10, 20, and 40 mg kg^−1^) by gavage, respectively; in the sepsis mouse model, the administration group mice were orally administered with either tirbanibulin (20, 40 mg kg^−1^), bosutinib (40, 80, and 160 mg kg^−1^), or dasatinib (20, 40, and 80 mg kg^−1^), respectively.

### In Vitro Drugs Intervention in Neutrophils

Neutrophils were treated with Src inhibitor (tirbanibulin), raf inhibitor (GW5074), or vehicle (dimethyl sulfoxide, 1% v/v) 30 min before PMA stimulation, and then as described above.

### Reagents and Antibodies

All reagents and antibodies involved in this study are shown in Tables S3 and S4 (Supporting Information).

### Histological Analysis

Pancreatic and lung tissues were fixed with 4% paraformaldehyde and embedded in paraffin, followed by hematoxylin‐eosin staining and optical microscopy examination. The histopathological score analysis of pancreatic and lung tissues was blindly conducted by two independent pathologists based on the previously described method.^[^ ^45^, ^46^ ^]^

### Neutrophil Preparation and Stimulation

Mouse bone marrow and human peripheral blood neutrophils were separated using a Percoll density gradient.^[^ ^14^ ^]^ Bone marrow‐derived neutrophils were isolated from the femur and tibia of euthanized mice using the mouse bone marrow‐derived neutrophil isolation kit under sterile conditions. Meanwhile, human peripheral blood neutrophils were isolated using the Lymphoprep Human lymphocyte isolate.

To evaluate NETs formation, the classic in vitro NETs model was induced by PMA. In brief, fresh isolated bone marrow neutrophils were seeded in 12‐well plates and cultured in Roswell Park Memorial Institute (RPMI)‐1640 medium containing 10% fetal bovine serum (FBS) at 37 °C in a 5% CO_2_ environment for 30 min, followed by treatment with 100 nm PMA or PBS for 4 h.

### Measurement of NETs Formation

To observe the formation of NETs in vitro, in short, bone marrow‐derived neutrophils were first fixed with 4% paraformaldehyde, followed by washing the samples with PBS, and then incubated overnight at 4 °C in the solution of anti‐MPO and anti‐citrulline histone H3 (Cith3) antibodies. Then placed the slides in PBS and washed three times, followed by incubation with the corresponding fluorescent secondary antibodies at 37 °C for 2 h. At last, incubated the slides in the 4,6‐diamino‐2‐phenyl indole (DAPI) solution at 37 °C for 5 min.

By observing the content of NETs formed in vivo, the pancreatic and lung tissues of mice were fixed, embedded, and cut into 5 µm thick slices. Then boiled the slices in an antigen repair buffer containing ethylenediamine tetraacetic acid and blocked the slides with normal goat serum. Next, incubated the slides with anti‐MPO and anti‐Cith3 antibodies overnight at 4 °C. Washed the slides three times and incubated these with the corresponding fluorescent secondary antibodies at 37 °C for 2 h. Then, the tissue slices were incubated in the DAPI solution at 37 °C.

Finally, images were obtained using the confocal microscope (Leica TCS Sp8 sted, Germany) and its corresponding LAS X imaging software.

### Enzyme Linked Immunosorbent Assay (ELISA) for Measuring Cytokines

In the light of the manufacturer's instructions, human interleukin‐6 (IL‐6) and tumor necrosis factor‐alpha (TNF‐α) ELISA kits were operated for detecting IL‐6 and TNF‐α concentration in human serum. Moreover, using a mouse IL‐6, TNF‐α, and monocyte chemotactic protein‐1 (MCP‐1) ELISA kit for measuring IL‐6, TNF‐α, and MCP‐1 concentration in mouse serum and cell supernatant.

### Detection of MPO‐DNA Complex

According to a previously described protocol,^[^ ^11^, ^47^ ^]^ 96‐well plates were coated with an anti‐human myeloperoxidase (MPO) antibody, diluted with PBS to 5 µg mL^−1^, overnight at 4 °C. The plate was then washed twice with washing buffer (0.05% Tween‐20 in PBS) and sealed at room temperature for 2 h with 4% bovine serum albumin (BSA) in PBS (0.05% Tween‐20 added). The plate was washed five times. Serum samples were added and incubated at room temperature for 2 h. The plate was washed five times before adding anti‐DNA antibodies (horseradish peroxidase (HRP) coupling; cell death kit) in blocking buffer at 1:100 and incubating at room temperature for 2 h. After washing the plate five times, it was incubated with 3,3′,5,5′‐Tetramethylbenzidine (TMB) substrate; the reaction was terminated with a termination solution (cell death kit), and the absorbance of each well was detected at a wavelength of 450 nm. The results were reported with the percentage of plasma in the control group ± standard deviation (SD), set to 100% in the control group.

### Quantitative Analysis of dsDNA

According to the manufacturer's instructions, quantified human blood dsDNA via using the Quant‐iT PicoGreen dsDNA Quantitative Kit and dsDNA Reagent (Invitrogen).

### Flow Cytometry Analysis

The expression of p‐Src (Tyr418) and MPO was evaluated in mouse‐derived cells by incubating cells with primary antibodies against the surface markers anti‐CD45.2 (clone 104), anti‐CD11b (clone M1/70), and anti‐Ly6G (clone 1A8) at 4 °C for 30 min. After fixation, membrane rupture, washing, and resuspension in PBS, cells were incubated with anti‐p‐Src (Tyr418) (clone EP503Y) or anti‐MPO (clone EPR20257) antibodies.

The main steps for detecting p‐Src (Tyr418) in human peripheral blood cells were as follows. Briefly, cells were incubated with primary antibodies against the surface markers anti‐CD45 (clone 2D1), anti‐CD11b (clone M1/70), and anti‐CD16 (clone 3G8) at 4 °C for 30 min. And then anti‐p‐Src (Tyr418) (clone REA434) antibody was stained after fixation, the membrane ruptured, and then washed and resuspended with PBS.

Cellular ROS levels were evaluated using dihydroehidium (DHE) fluorescent probes. That is, cells were incubated with DHE solution in the dark at 37 °C for 30 min. Cells were washed with PBS, stained with the above‐described surface antibodies at 4 °C for 30 min, washed, and resuspended in PBS. The detailed method overview was provided in the previous study.^[^ ^46^ ^]^

Cells were processed on a Beckman DxFlex and assessed with CytExpert software.

### Scanning Electron Microscopy (SEM) Observation

In brief, treated neutrophils were fixed with 2.5% glutaraldehyde at 4 °C for 24 h. After standard dehydration and sputtering coating processes, the formation of NETs was observed using the scanning electron microscope (GeminiSEM 300, Britain).

### In Vitro Cell Line Culture and Transfection

HEK‐293T cells were purchased from Procell Life Science and Technology Co., Ltd. (Wuhan, China) and cultured in Dulbecco's modified Eagle's medium (DMEM) containing 10% FBS and 1% penicillin‐streptomycin in a humid atmosphere at 37 °C and 5% CO_2_. Cells were transfected with full‐length RAF1 (WT), RAF1 (Ser621A), RAF1 (Ser621D), RAF1 (Ser619A), and RAF1 (Ser621D) plasmid constructs (Obio Technology Co., Ltd., Shanghai, China) with Lipofectamine 3000 transfection reagent according to the manufacturer's instructions. The pcDNA3.1 plasmid was used for control transfection. Cells were harvested 48 h after transfection for further analysis.

### Western Blot Analysis and Co‐Immunoprecipitation (Co‐IP)

Total protein was extracted from cells using the radio immunoprecipitation (RIPA) assay buffer containing protease inhibitors. The protein concentration was measured using a bicinchoninic acid (BCA) protein assay method according to the manufacturer's instructions.

For western blotting, proteins were separated via sodium dodecyl‐sulfate polyacrylamide gel electrophoresis (SDS‐PAGE), transferred to a polyvinylidene fluoride (PVDF) membrane, blocked with 5% BSA to prevent non‐specific binding, and incubated with the following primary antibodies at 4 °C overnight: anti‐RAF1, anti‐RAF1 (phospho S621), anti‐RAF1 (phospho S259), anti‐RAF1 (phospho S301), anti‐Src, anti‐Src (photo Y418), anti‐phospho‐ERK (Thr202/Tyr204), anti‐ERK, anti‐phospho‐MEK (Ser217/221), anti‐MEK and anti‐β‐actin. Subsequently, the membranes were washed thrice with tris‐buffered saline solution with Tween‐20 (TBST) for 15 min each and incubated with an appropriate HRP‐coupled second antibody at 37 °C for 2 h. After washing, the ECL‐Plus chemiluminescence system detected the protein bands, and ImageJ software was used to analyze image intensity.

For Co‐IP, the Pierce Cross‐linked Magnetic IP/Co‐IP Kit was used according to the manufacturer's instructions. The antibodies used were as follows: anti‐RAF1, anti‐Src (photo Y418), anti‐rabbit mAb IgG XP isotype Control (Sepharose Bead Conjugate), anti‐Src, DYKDDDDK tag Polyclonal antibody (Binds to FLAG tag epitope), and anti‐β‐actin. All collected protein lysates were eluted with 6 × SDS loading buffer and boiled at 100 °C for 10 min. Following elution, SDS‐PAGE was performed.

### Immunoprecipitation‐Mass Spectrometry (IP/MS) Analysis and Protein Identification

After Co‐IP, the SDS‐PAGE gel was loaded with the same amount of protein. According to the manufacturer's instructions, Pierce silver stain for mass spectrometry was performed. Subsequently, the 75–50 kDa bands were cut into separate fragments, excluding the stained IgG. The fragments were cut into smaller pieces and placed in tubes. Samples for the Q‐Exactive ^Plus^ mass spectrometer were prepared according to a standard method.^[^ ^48^ ^]^ After the gel was decolorized and shrunken, the proteins were reduced and digested with the enzymolysis working solution. Finally, IP/MS protein identification analysis was conducted by Shanghai OE Biotech Co., Ltd. (Shanghai, China).

### RNA Isolation and Reverse Transcription Polymerase Chain Reaction (PCR)

Total RNA was extracted from cells using 1 mL of TRIzol reagent. The cell lysates were then homogenized on ice, allowed to stand for 5 min, and mixed with 0.1 × bromocresolpuple. After 15—20 s of vigorous shaking, the lysates were left to stand at room temperature for 5 min, and then centrifuged at 12 000 × g and 4 °C for 15 min. The supernatant was aspirated into a new tube, and an equal volume of isopropanol was added, mixed thoroughly, and precipitated at −80 °C for 30 min. Subsequently, the samples were centrifuged at 12 000 × g and 4 °C for 15 min, washed twice with 75% ethanol, air dried, and mixed with an appropriate amount of ribonuclease‐free water to dissolve RNA. All samples were subjected to real‐time quantitative PCR using standardized 18S gene expression to quantify relative mRNA levels. The primer sequences used to amplify the mRNAs are listed in Table S5 (Supporting Information).

### RNA‐Seq Analysis

RNA‐seq was used to analyze the mRNA expression in PMA‐induced NET formation. In brief, total RNA was extracted from mouse bone marrow neutrophils in the S100a8^cre^ Src^fl/fl^ + PMA and Src^fl/fl^ + PMA groups using TRIzol universal reagent. The mRNA in the two groups was extracted by Novogene Co., Ltd. (Tianjin, China) and used for RNA‐seq analysis. Subsequently, data analysis was conducted using R software to identify differentially expressed genes and related enrichment pathways between the two groups.

### Statistical Analysis

Statistical analysis was performed in the GraphPad Prism 8.0 software (GraphPad, San Diego, CA, USA). The variable correlation heatmap analysis was conducted using the R language software. An independent sample *t*‐test was used to analyze the mean values between two groups, and one‐way analysis of variance (ANOVA) was used to evaluate the statistically significant differences between all groups. The survival curve was obtained using the Kaplan–Meier method and compared using logarithmic rank test. The results were expressed as mean ± standard error (SE), and *P* < 0.05 was considered statistically significant (double‐tailed). Repeated each experiment at least 2 times.

## Conflict of Interest

The authors declare no conflict of interest.

## Author Contributions

G.L., F.H., Y.W., and C.Y. contributed equally to this work. F.H., L.C., C.Y., and T.X. provided animals and cells data. G.L. and Y.W. wrote and reviewed the manuscript. Q.Z., Y.W., and X.D. analyzed and interpreted the data (statistical analysis, biostatistics, computational analysis). Y.D., W.X., Y.Z., J.P., B.T., W.L., H.X., and W.C. acquired the data (e.g., acquired and managed patients, provided facilities). G.L., W.G., F.W., and L.H. conceived and designed the study, revised the manuscript and supervised the study.

## Supporting information



Supporting Information

## Data Availability

The RNA sequencing data can be downloaded from the NCBI GEO accession code GEO: GSE251972. All original data has been deposited at NCBI GEO and is publicly available as of the date of publication. The code used in this study are all open access R packages in R language software, which have been described in the statistical analysis section, and there are no restrictions to availability.
