# Supplementary material for: Src Reduces Neutrophil Extracellular Traps Generation and Resolves Acute Organ Damage
Source: Adv Sci (Weinh). 2025 Aug 26;12(41):e06028. doi: 10.1002/advs.202506028 (PMC12591175; doi:10.1002/advs.202506028)
Supplement: Supplementary file 1 — Supporting Information [file ADVS-12-e06028-s001.docx]

**Supplemental Information**

**
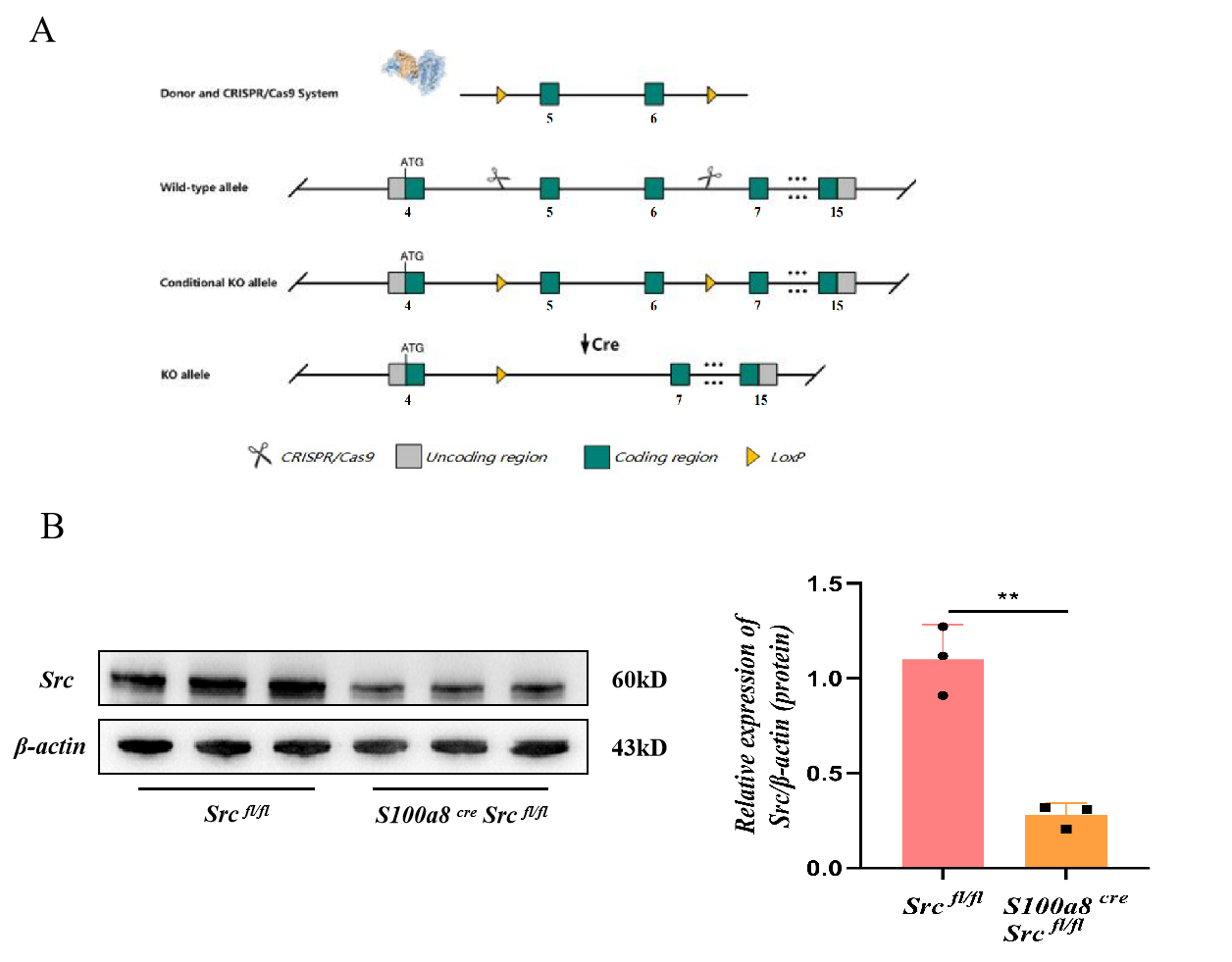
**

**Supplemental Figure 1. Construction of S100a8^cre^Src^fl/fl^ mice using CRISPR/Cas9 technology.** (A) The specific breeding scheme of S100a8^cre^ Src^fl/fl^ mice. (B) Src protein abundance determined via w[estern blotting](https://www.sciencedirect.com/topics/biochemistry-genetics-and-molecular-biology/western-blot) *(n* =3). Relative protein expression of Src; β-actin is used as control for protein loading. (*n* = 3).


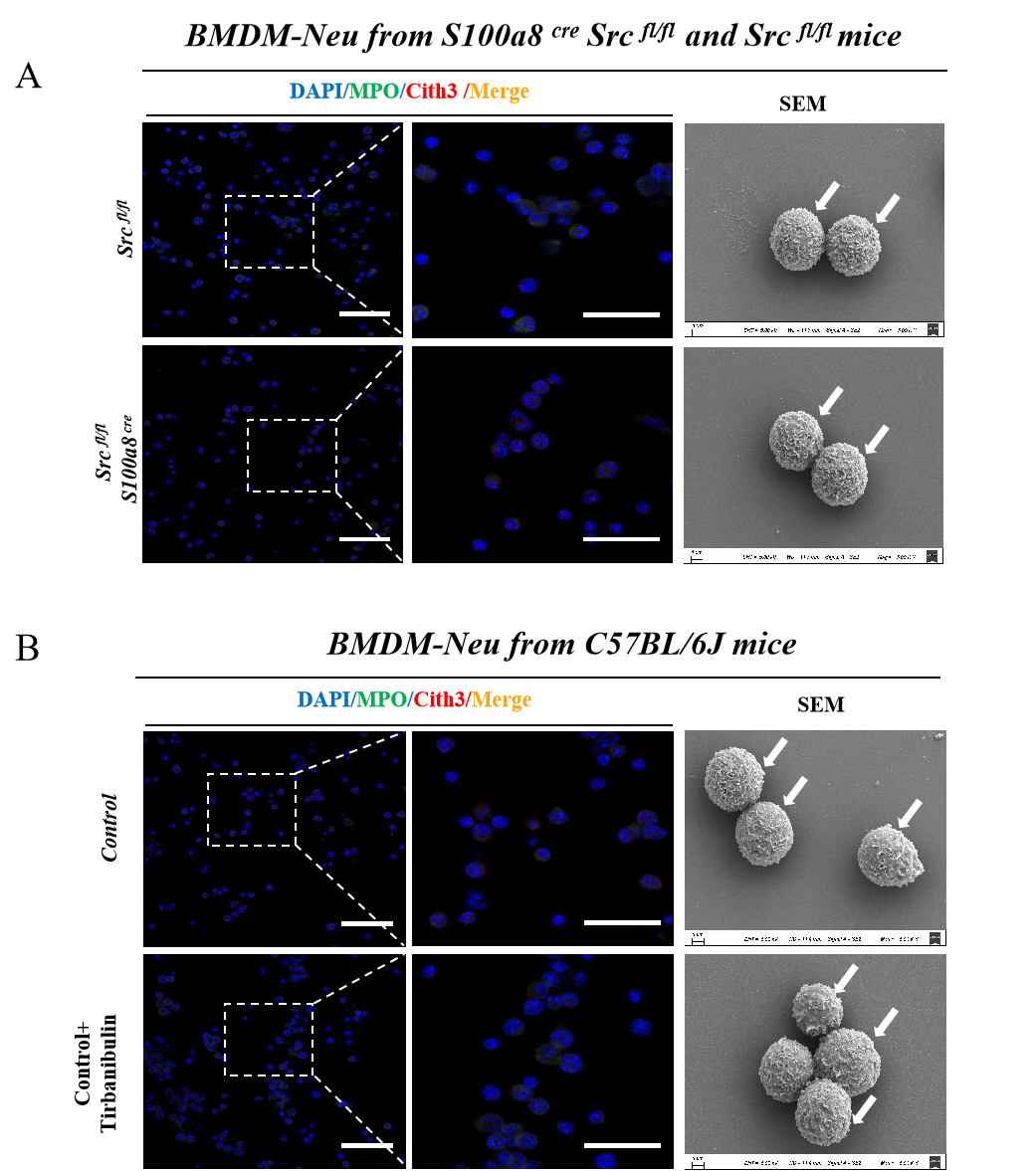


**Supplemental Figure 2. Representative immunofluorescence images of Cith3 and MPO and representative scanning electron microscopy of neutrophils.** (A) Representative immunofluorescence image of Cith3 and MPO of neutrophils in two groups (Src^fl/fl^ and S100a8^cre^ Src^fl/fl^) at 400× (Scale Bar = 50 μM) and 1000× (Scale Bar = 10 μM) magnification. Representative SEM of neutrophils in two groups (Src^fl/fl^ and S100a8^cre^ Src^fl/fl^) . White arrows: neutrophils, yellow arrows: NETs (*n* = 6). (B) Representative immunofluorescence image of Cith3 and MPO of neutrophils in two groups (Control and Control+Tirbanibulin) at 400× (Scale Bar = 50 μM) and 1000× (Scale Bar = 10 μM) magnification. Representative SEM of neutrophils in two groups (Control and Control+Tirbanibulin) . White arrows: neutrophils, yellow arrows: NETs (*n* = 6).


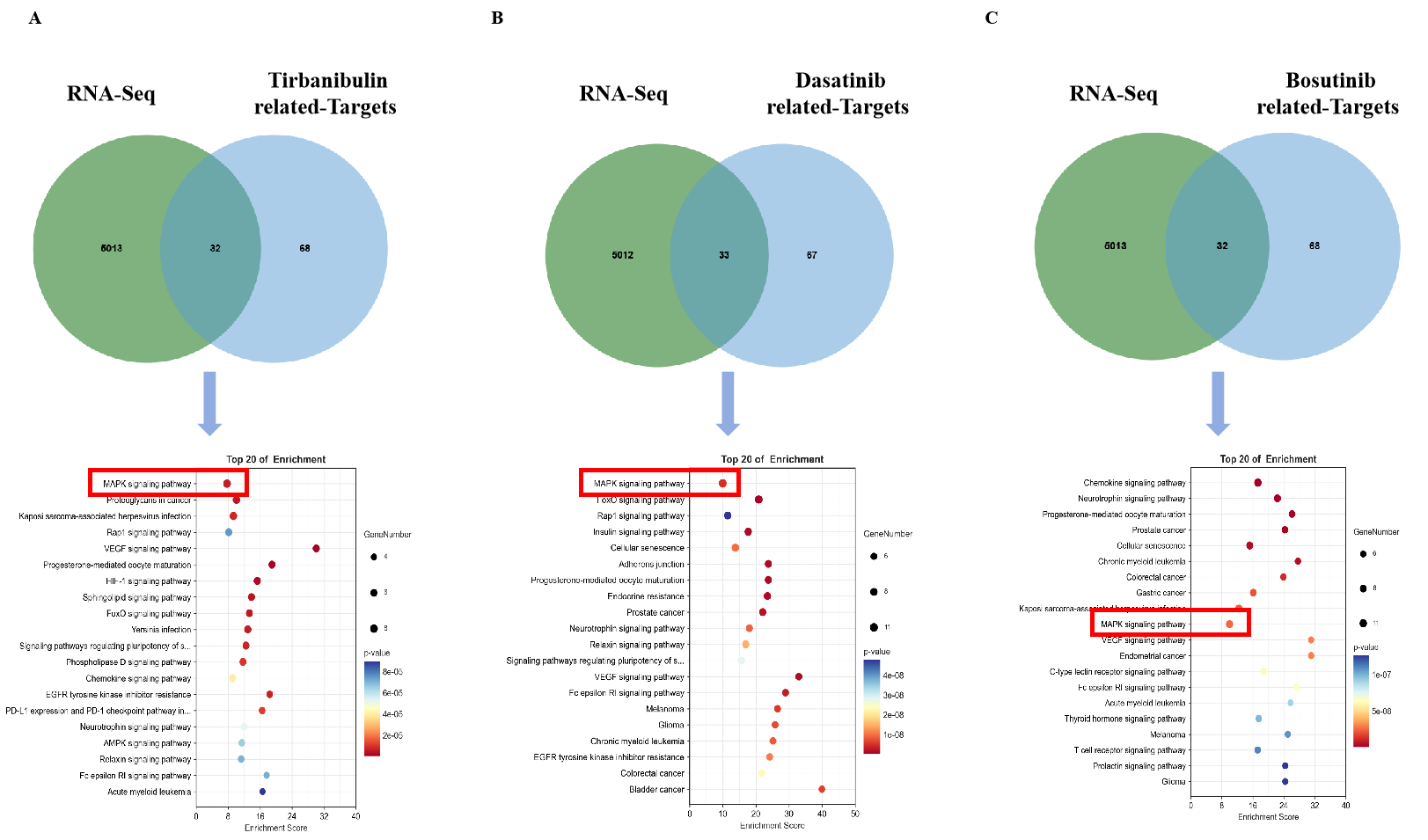


**Supplemental Figure 3. The SwissTarget Prediction website predicts the targets of Src inhibitors and combines them with the RNA-Seq data (RNA-Seq analysis of PMA induced S100a8^cre^ Src^fl/fl^ mice and Src^fl/fl^ mice bone marrow neutrophils) to analyze enriched pathways.** (A) The SwissTarget Prediction website predicts the targets of Src inhibitor (Tirbanibulin). Venn diagram of core targets screened by combined analysis of Tirbanibulin targets and RNA-Seq, and KEGG enrichment analysis of the differential core targets. (B) The SwissTarget Prediction website predicts the targets of Src inhibitor (Dasatinib). Venn diagram of core targets screened by combined analysis of Dasatinib targets and RNA-Seq, and KEGG enrichment analysis of the differential core targets. (C) The SwissTarget Prediction website predicts the targets of Src inhibitor (Bosutinib). Venn diagram of core targets screened by combined analysis of Bosutinib targets and RNA-Seq, and KEGG enrichment analysis of the differential core targets.


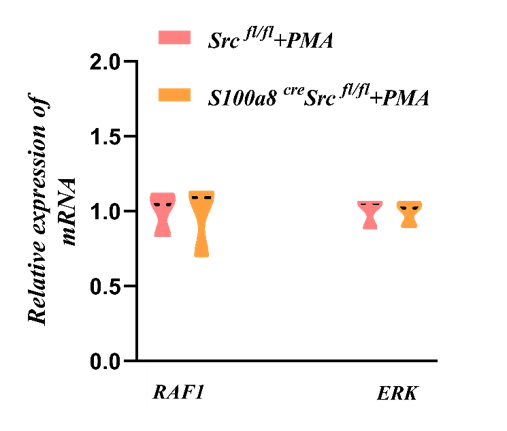


**Supplemental Figure 4. The mRNA levels of RAF1 and ERK in S100a8cre Srcfl/fl + PMA and Srcfl/fl + PMA neutrophils (n =3).**


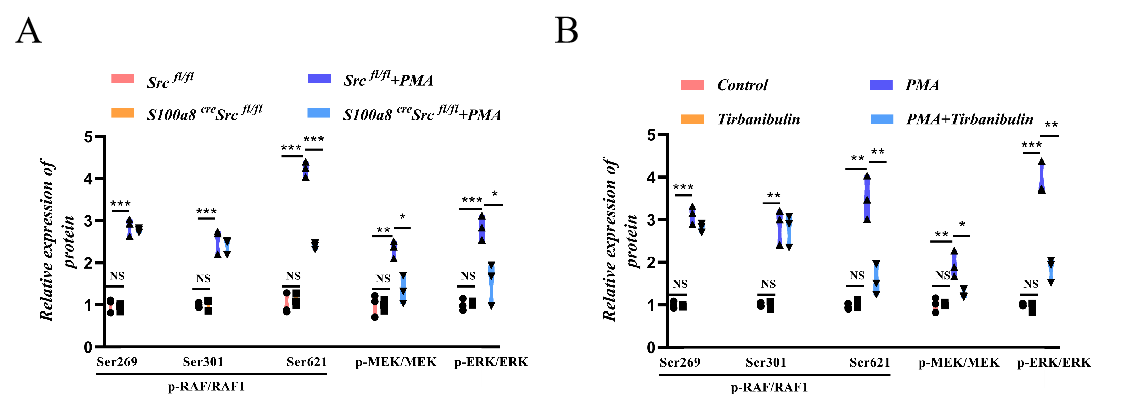


**Supplemental Figure 5. Relative protein expression of p-RAF1, p-MEK and p-ERK in neutrophils.** (A) Relative protein expression of p-RAF1, p-MEK and p-ERK in neutrophils of four groups (Src^fl/fl^, S100a8^cre^Src^fl/fl^, PMA+Src^fl/fl^, PMA+S100a8^cre^Src^fl/fl^) (n = 3); RAF1, MEK and ERK are used as control for protein loading (n = 3). (B) Relative protein expression of p-RAF1, p-MEK and p-ERK in neutrophils of four groups (Control, Control + Tirbanibulin, PMA, PMA + Tirbanibulin) (n = 3); RAF1, MEK and ERK are used as control for protein loading (n = 3). Statistical significance was denoted as:^*^*P < 0.05*, ^**^*P < 0.01*, ^***^*P < 0.001*.

**
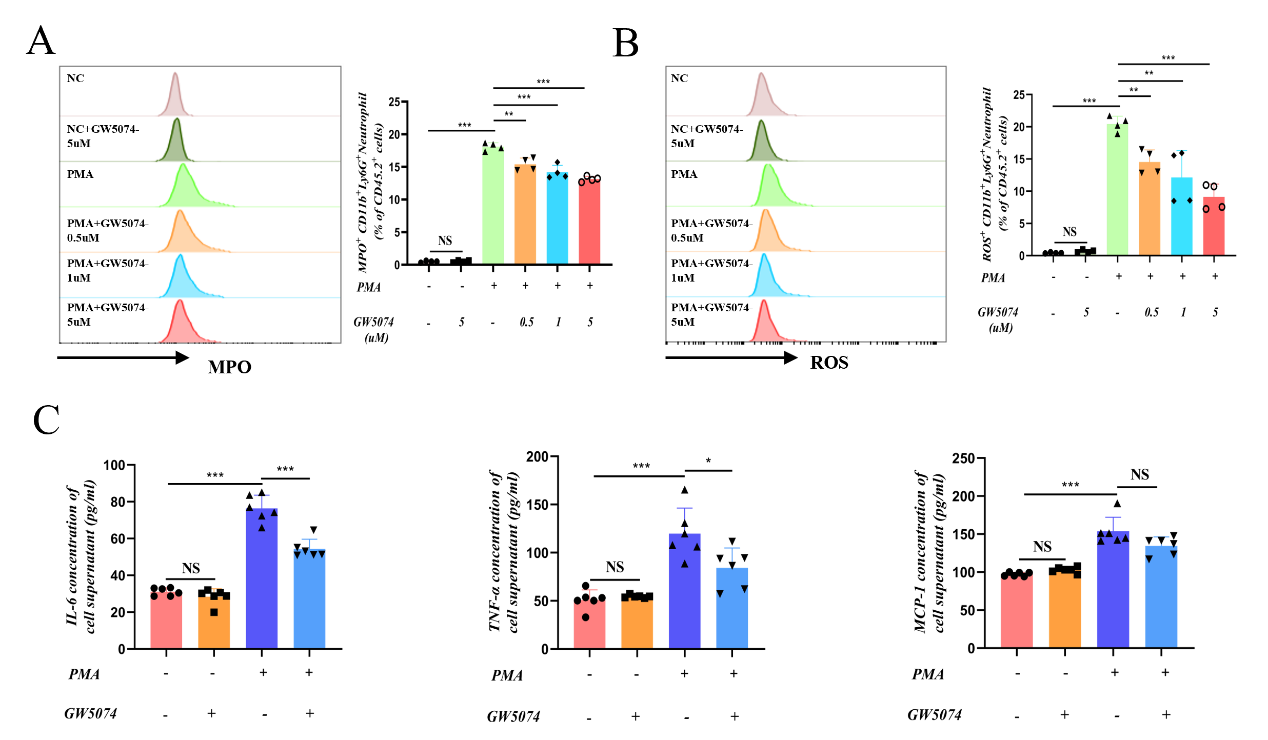
**

**Supplemental Figure 6. RAF inhibitor stifles NETs formation.** (A-B) Bone marrow neutrophils isolated from C57BL/6J mice are cultured and stimulated with PMA (100 nM), and incubated with different doses of GW5074 (0.5, 1, and 5 um) before flow cytometry analysis. Representative flow cytometry gating of bone marrow neutrophils (CD45.2^+^ CD11b^+^ Ly6G^+^). Representative flow cytometry plots and bar graphs depicting the proportion of neutrophils and the expression levels of MPO and ROS. Data is presented as mean ± SD (*n* = 4). (C) Supernatant levels of IL6, TNF-α, and MCP-1 detected using ELISA; (*n* = 6). Statistical significance was denoted as: ^*^*P < 0.05*, ^**^*P < 0.01*, ^***^*P < 0.001*.


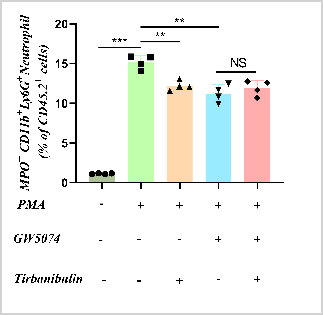


**Supplemental Figure 7. Representative bar charts of MPO expression level in five groups: Control, PMA, PMA+Tirbanibulin, PMA+GW5074, and PMA+Tirbanibulin+GW5074.** Data is presented as mean ± SD (N=4). Statistical significance was denoted as: ^**^*P < 0.01*, ^***^*P < 0.001*.


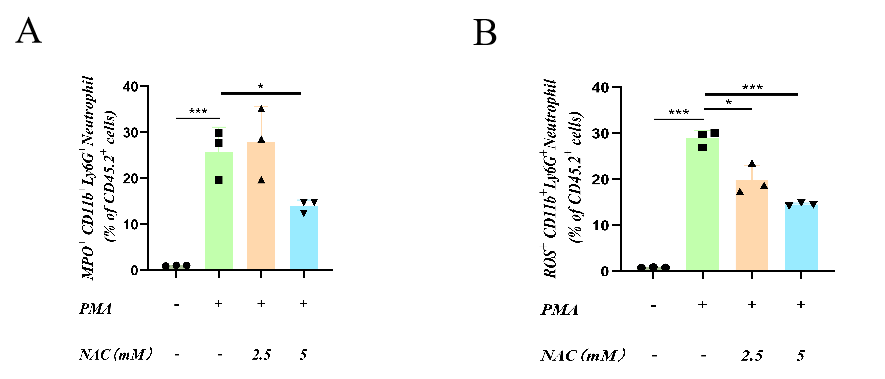


**Supplemental Figure 8. ROS inhibitor suppresses NETs formation.** (A-B) Bone marrow neutrophils isolated from C57BL/6J mice are cultured and stimulated with PMA (100 nM), and incubated with different doses of ROS inhibitor(NAC) (2.5 and 5 mM) before flow cytometry analysis. Representative flow cytometry gating of bone marrow neutrophils (CD45.2^+^ CD11b^+^ Ly6G^+^). Representative flow cytometry plots and bar graphs depicting the proportion of neutrophils and the expression levels of MPO and ROS. Data is presented as mean ± SD (*n* = 3). Statistical significance was denoted as: ^*^*P < 0.05*, ^***^*P < 0.001*.


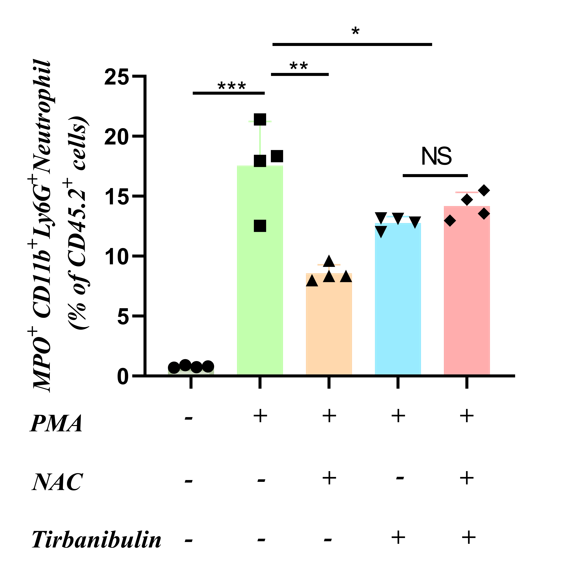


**Supplemental Figure 9. Representative bar charts of MPO expression level in five groups: Control, PMA, PMA+Tirbanibulin, PMA+NAC, and PMA+Tirbanibulin+NAC.** Data is presented as mean ± SD (N=4). Statistical significance was denoted as: ^*^*P < 0.05*, ^**^*P < 0.01*, ^***^*P < 0.001*.


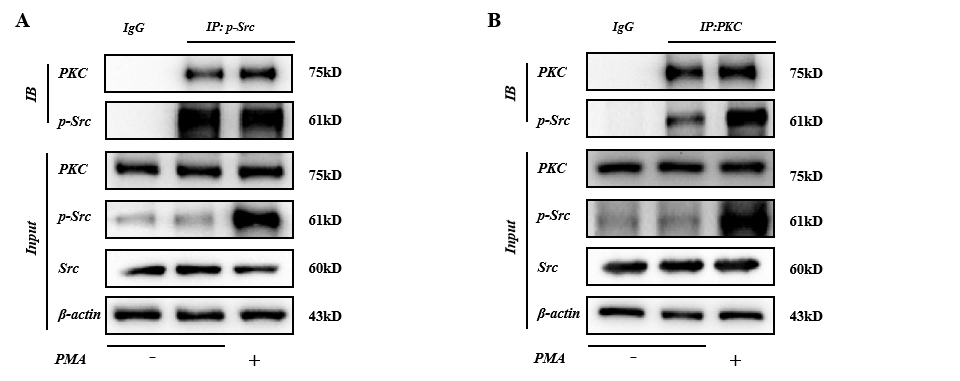


**Supplemental Figure 10. Co-IP of the interaction between p-Src and PKC. Neutrophil lysates isolated from the bone marrow of C57BL/6J mice assessed by Co-IP with anti-Src Family (photo Y418) antibody or anti-PKC antibody.**

**
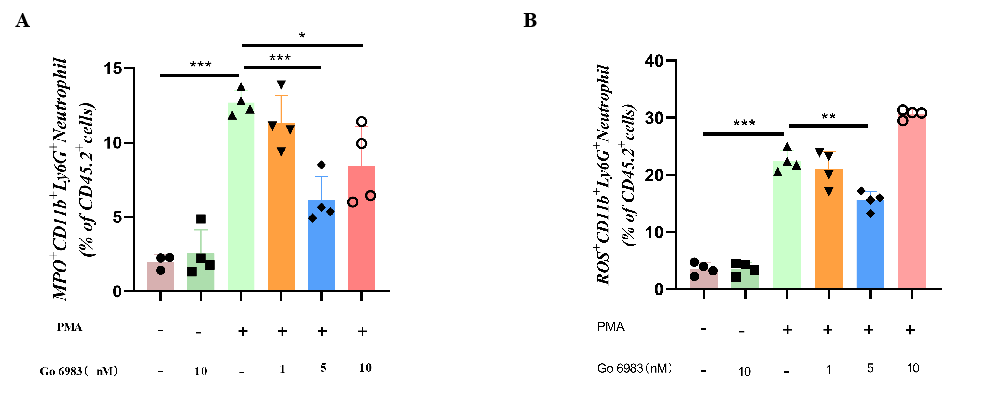
**

**Supplemental Figure 11. PKC inhibitor inhibits NETs formation.** (A-B) Bone marrow neutrophils isolated from C57BL/6J mice are cultured and stimulated with PMA (100 nM), and incubated with different doses of PKC inhibitor (Go 6983) (1, 5 and 10 nM) before flow cytometry analysis. Representative flow cytometry gating of bone marrow neutrophils (CD45.2^+^ CD11b^+^ Ly6G^+^). Representative flow cytometry plots and bar graphs depicting the proportion of neutrophils and the expression levels of MPO and ROS. Data is presented as mean ± SD (n = 3). Statistical significance was denoted as: ^*^*P < 0.05*, ^**^*P < 0.01*, ^***^*P < 0.001*.

**
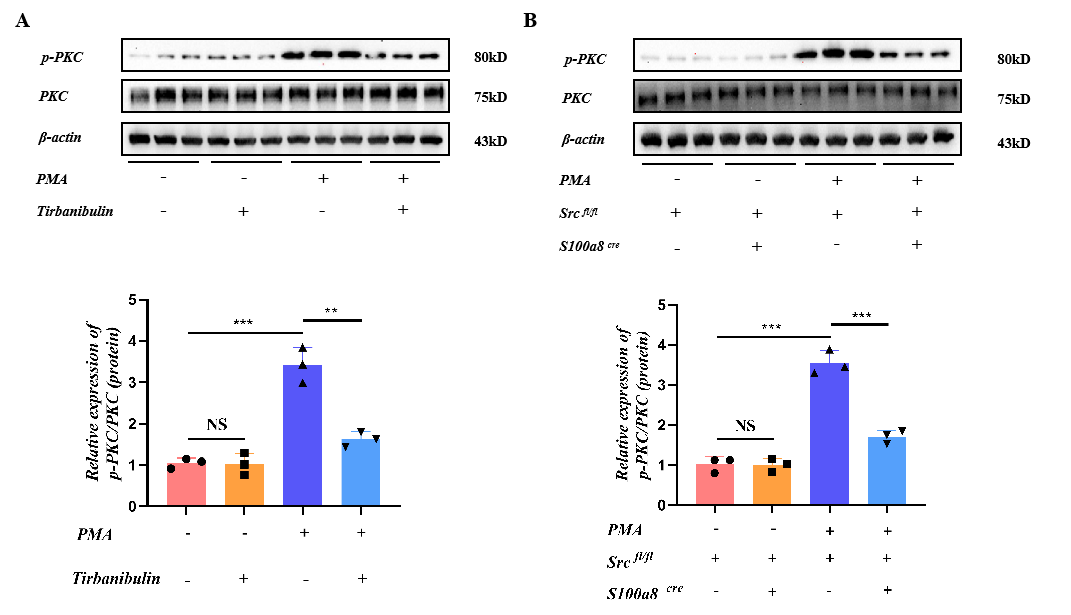
**

**Supplemental Figure 12. The protein levels of p-PKC in neutrophils of Src^fl/fl^, S100a8^cre^ Src^fl/fl^, PMA+Src^fl/fl^, and PMA+ S100a8^cre^ Src^fl/fl^ groups determined by western blotting (N = 3). The protein levels of p-PKC in neutrophils of four groups (Control, Control + Tirbanibulin, PMA, PMA + Tirbanibulin) assessed using w**[**estern blotting**](https://www.sciencedirect.com/topics/biochemistry-genetics-and-molecular-biology/western-blot) **(N = 3).**


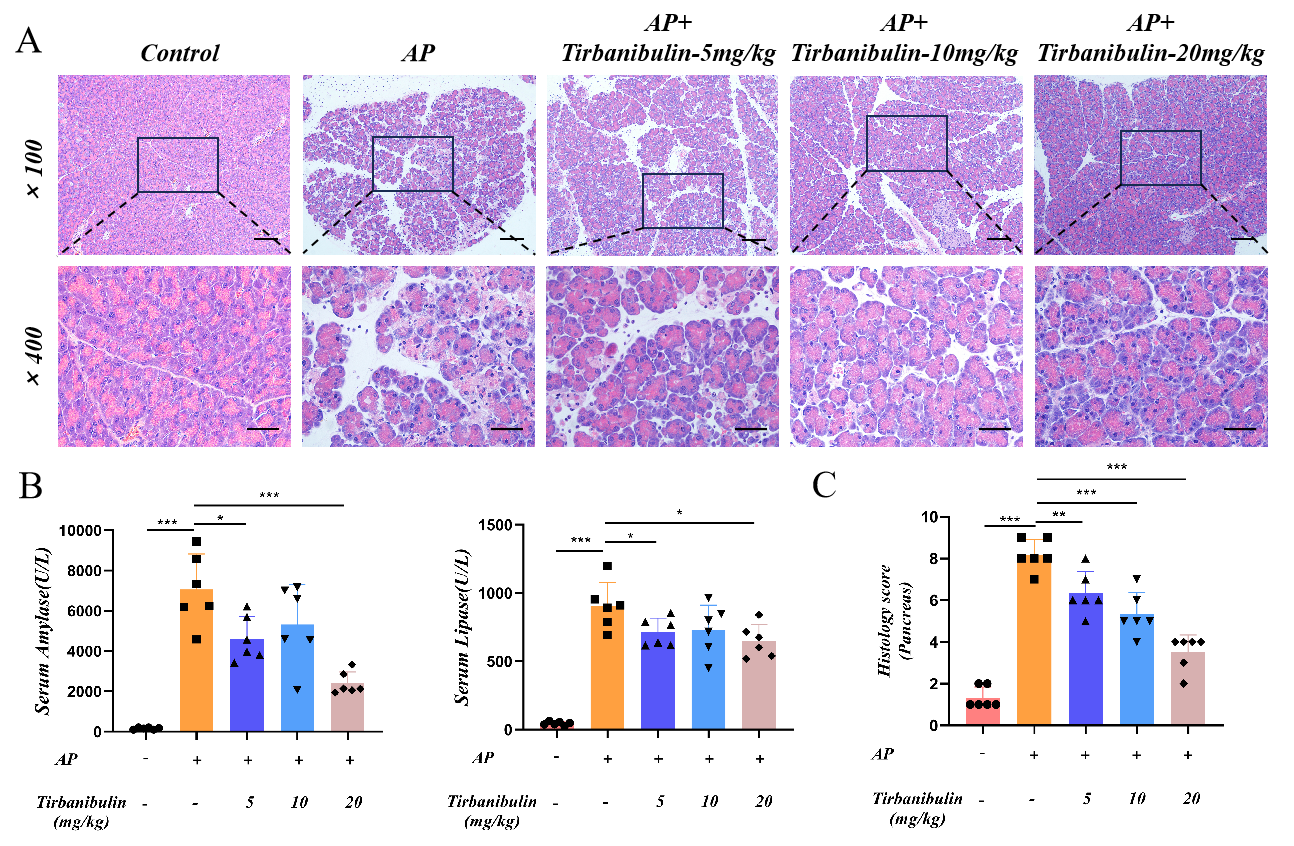


**Supplemental Figure 13. Tirbanibulin ameliorates the severity of AP mice.** (A) Representative H&E staining of pancreatic tissues at 100× (Scale Bar = 100 μM) and 400× (Scale Bar = 50 μM) magnification (*n* = 6). (B) Serum levels of amylase and lipase (*n* = 6). (C) Pathological scores of pancreatic tissues (*n* = 6). Statistical significance was denoted as: ^*^*P < 0.05*, ^**^*P < 0.01*, ^***^*P < 0.001*.


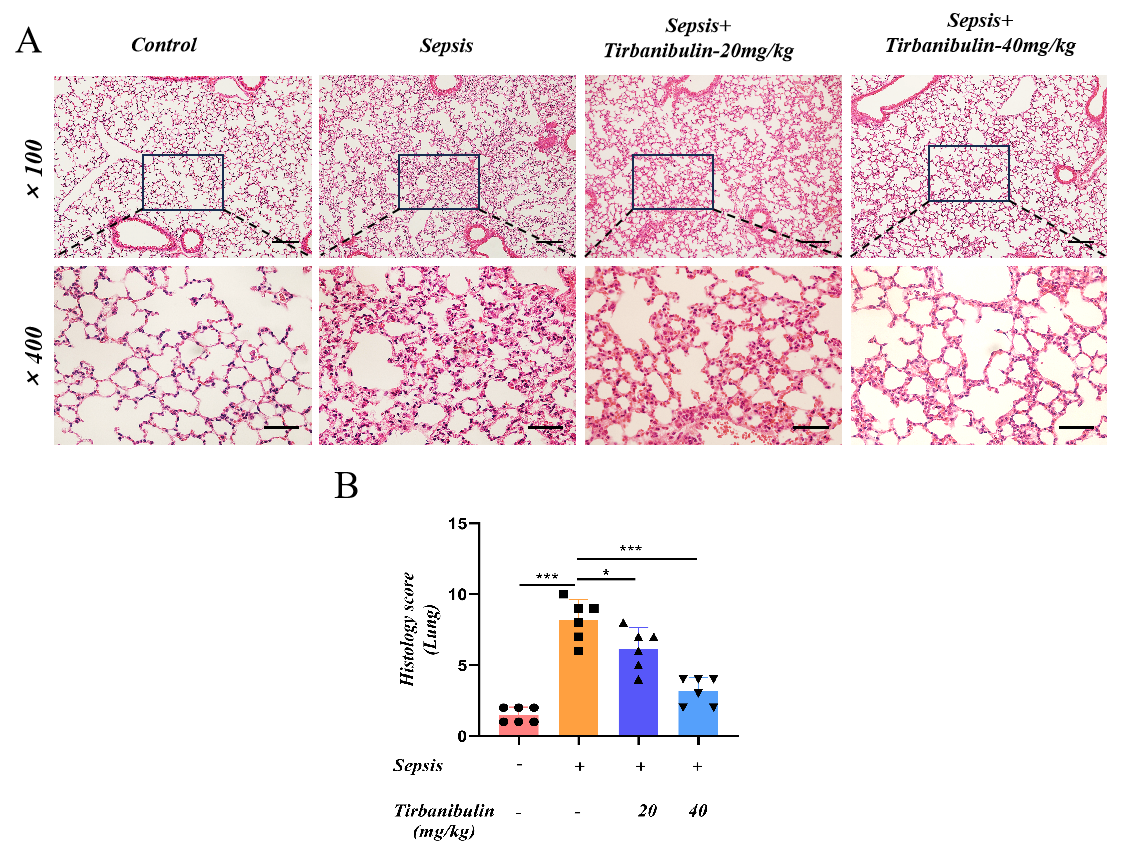


**Supplemental Figure 14. Tirbanibulin ameliorates the severity of Sepsis mice.** (A) Representative H&E staining of lung tissues at 100× (Scale Bar = 100 μM) and 400× (Scale Bar = 50 μM) magnification (*n* = 6). (B) Pathological scores of pancreatic tissues (*n* = 6). Statistical significance was denoted as: ^*^*P < 0.05*, ^***^*P < 0.001*.

**
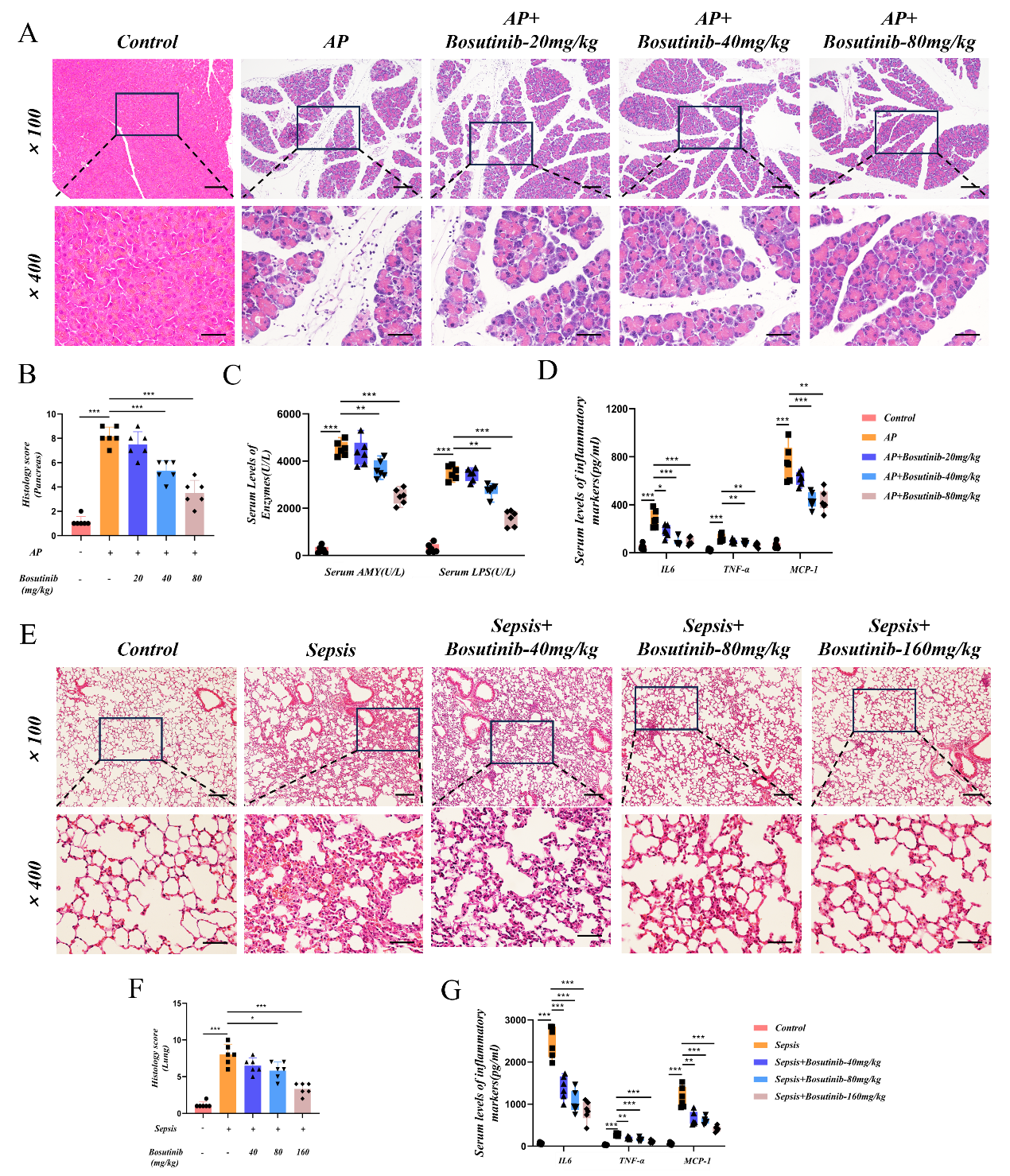
**

**Supplemental Figure 15. Bosutinib alleviates the severity of AP and sepsis.** (A) Representative H&E staining of pancreatic tissues at 100× (Scale Bar = 100 μM) and 400× (Scale Bar = 50 μM) magnification (*n* = 6). (B-C) Serum levels of amylase and lipase (*n* = 6). (D) Pathological scores of pancreatic tissues (*n* = 6). (E-G) Serum levels of IL6, TNF-α, and MCP-1 are detected using ELISA (*n* = 6 per group). (H) Representative H&E staining of lung tissues at 100× (Scale Bar = 100 μM) and 400× (Scale Bar = 50 μM) magnification (*n* = 6). (I) Pathological scores of lung tissues (*n* = 6). (J-L) Serum levels of IL6, TNF-α, and MCP-1 are detected using ELISA (*n* = 6). Statistical significance was denoted as: ^*^*P < 0.05*, ^**^*P < 0.01*, ^***^*P < 0.001*.

**
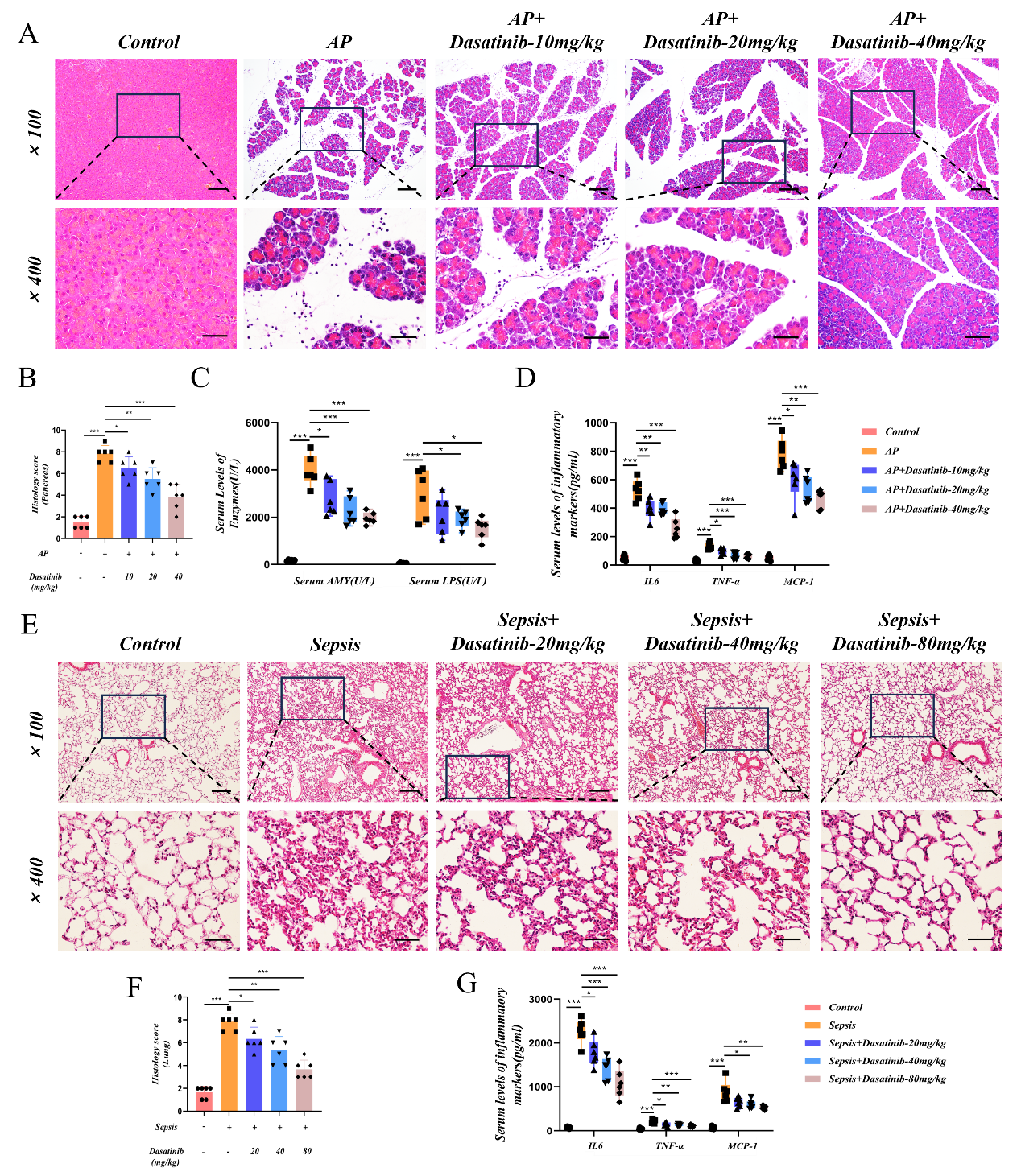
**

**Supplemental Figure 16. Dasatinib mitigates the severity of AP and sepsis.** (A) Representative H&E staining of lung tissues at 100× (Scale Bar = 100 μM) and 400× (Scale Bar = 50 μM) magnification (*n* = 6). (B-C) Serum levels of amylase and lipase (*n* = 6). (D) Pathological scores of pancreatic tissues (*n* = 6). (E-G) Serum levels of IL6, TNF-α, and MCP-1 are detected using ELISA (*n* = 6 per group). (H) Representative H&E staining of lung tissues at 100× (Scale Bar = 100 μM) and 400× (Scale Bar = 50 μM) magnification (*n* = 6). (I) Pathological scores of lung tissues (*n* = 6). (J-L) Serum levels of IL6, TNF-α, and MCP-1 are detected using ELISA (*n* = 6). Statistical significance was denoted as: ^*^*P < 0.05*, ^**^*P < 0.01*, ^***^*P < 0.001*.


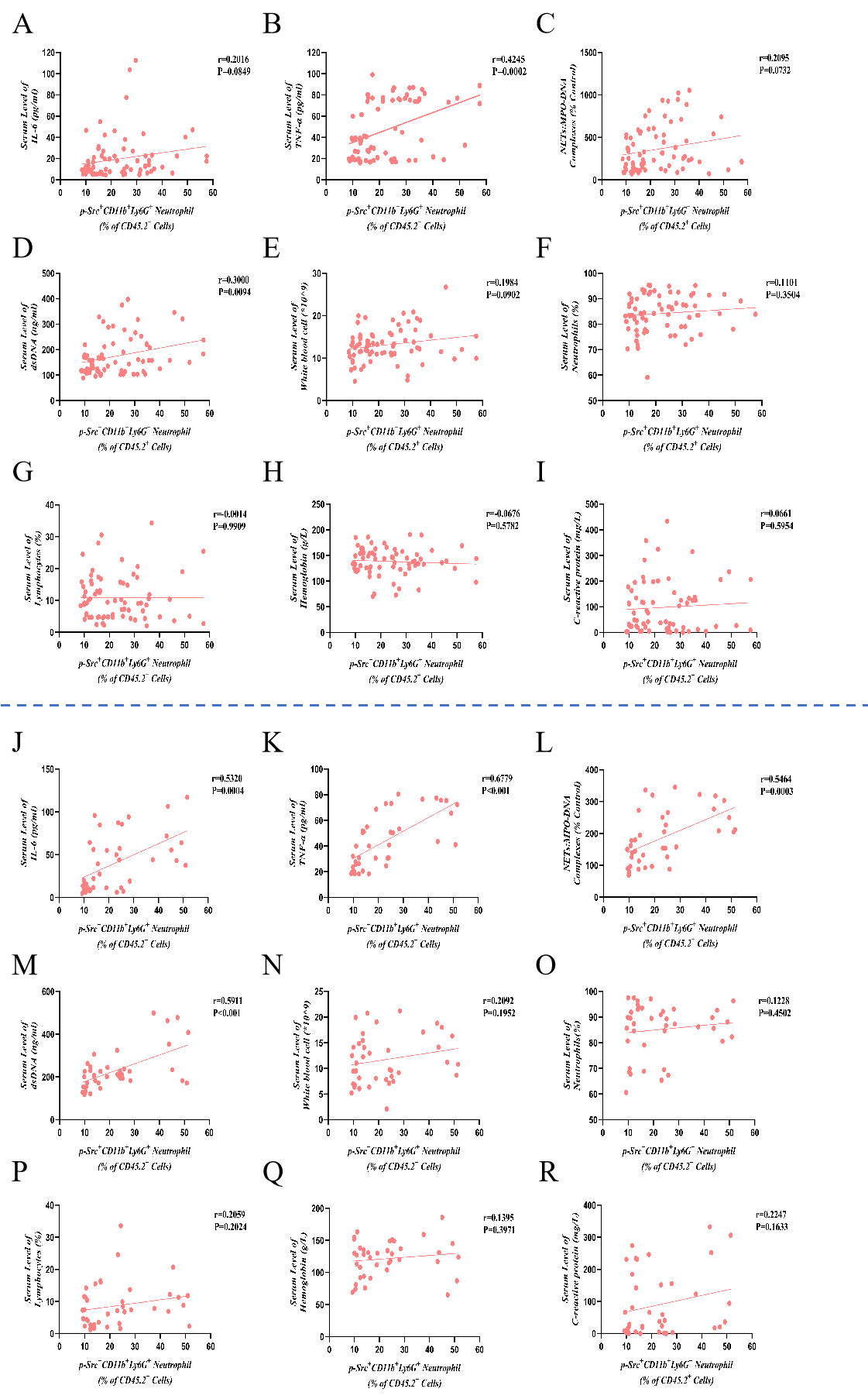


**Supplemental Figure 17. Correlation analysis between peripheral blood neutrophil p-Src levels and other clinical indicators in patients with AP and sepsis.** (A-I) Pearson correlation analysis of the expression level of neutrophil p-Src in peripheral blood and related serum clinical indicators of AP patients (IL6, TNF-α, MPO-DNA, dsDNA, white blood cell, neutrophils, lymphocytes, hemoglobin and c-reactive protein). (J-R) Pearson correlation analysis of the expression level of neutrophil p-Src in peripheral blood and related serum clinical indicators of sepsis patients (IL6, TNF-α, MPO-DNA, dsDNA, white blood cell, neutrophils, lymphocytes, hemoglobin and c-reactive protein).

**Supplemental Table 1. Demographic data and clinical characteristics of AP patients with or without LC.**

| ***Variables*** | ***Non-LC***  ***n=41*** | ***LC***  ***n=33*** | ***P value*** |
| --- | --- | --- | --- |
| Age, year (mean ± SD)  Male sex, N (%)  Smoking, N (%)  Drinking, N (%)  Hyperlipidemia, N (%)  hypertension, N (%)  Diabetes, N (%)  Fatty liver, N (%)  ***Clinical characteristics***  Leukocytes, *10^9/L (mean± SD)  Neutrophil, % (mean± SD)  Lymphocyte, % (mean± SD)  CRP, mg/L (mean± SD) | 45.39±13.58  24(58.5%)  14(34.1%)  11(26.8%)  16(39.0%)  11(26.8%)  7(17.1%)  13(31.7%)  12.21±3.37  82.22±6.01  11.36±4.94  72.07±84.00 | 48.88±14.63  22(66.7%)  10(30.3%)  10(30.3%)  15(45.5%)  9(27.3%)  13(39.4%)  12(36.4%)  14.23±4.41  86.69±7.91  10.44±9.09  127.89±110.09 | 0.292  0.949  0.730  0.746  0.583  0.967  0.038^*^  0.679  0.028^*^  0.007^**^  0.606  0.022^*^ |

Data was described as the mean ± SD, N (%). ^*^P<0.05, ^**^P<0.01.

AP, acute pancreatitis; LC, local complications; CRP, c-reactive protein.

**Supplemental Table 2. Demographic data and clinical characteristics of Sepsis patients with or without Survivors.**

| ***Variables*** | **Survivors**  ***n=23*** | ***Non* -Survivors**  ***n=17*** | ***P value*** |
| --- | --- | --- | --- |
| Age, year (mean ± SD)  Male sex, N (%)  Smoking, N (%)  Drinking, N (%)  Hyperlipidemia, N (%)  hypertension, N (%)  Diabetes, N (%)  Fatty liver, N (%)  ***Clinical characteristics***  Leukocytes, *10^9/L (mean± SD)  Neutrophil, % (mean± SD)  Lymphocyte, % (mean± SD)  CRP, mg/L (IQR) | 59.91±16.09  14(60.9%)  8(34.8%)  6(26.1%)  2(8.7%)  11(47.8%)  7(30.4%)  4(17.4%)  10.50±5.16  82.48±12.22  6.87±5.87  20.05(3.31,81.27) | 67.59±15.37  10(58.8%)  5(29.4%)  5(29.4%)  4(23.5%)  8(47.1%)  2(11.8%)  3(17.6%)  13.34±3.86  88.77±5.04  10.48±6.05  94.05(28.47,233.07) | 0.137  0.689  0.728  0.822  0.235  0.633  0.038^*^  0.984  0.064  0.033^*^  0.066  0.045^*^ |

Data was described as the mean ± SD, N (%), median (IQR). ^*^P<0.05.

CRP, c-reactive protein.

**Supplemental Table 3. Reagent tables.**

| **REAGENT or RESOURCE** | **SOURCE** | **IDENTIFIER** |
| --- | --- | --- |
| Tirbanibulin | MCE | HY-10340 |
| Caerulein | Sigma Aldrich | C9026 |
| Dihydroethidium (DHE) | Sigma Aldrich | D70N8 |
| Phorbol-12-myristate-13-acetate (PMA) | MCE | HY-18739 |
| GW5074 | TOPSCIENCE | T6525 |
| TRNzol Universal Reagent | TIANGEN | DP424 |
| Amylase kit | BioSino BioTechnology & Science | 100000060 |
| Lipase kit | Nanjing Jiancheng | A054-1-1 |
| Aspartate aminotransferase Assay Kit | Nanjing Jiancheng | C010-2-1 |
| Alanine aminotransferase Assay Kit | Nanjing Jiancheng | C009-2-1 |
| Mouse IL-6 Uncoated ELISA Kit | Affymetrix eBioscience | 88-7064-77 |
| Mouse TNF-α Uncoated ELISA Kit | Affymetrix eBioscience | 88-7324-77 |
| Mouse MCP-1 Uncoated ELISA Kit | Affymetrix eBioscience | 88-7391-77 |
| IL-6 Human Uncoated ELISA Kit | Affymetrix eBioscience | 88-7066-88 |
| TNF alpha Human Uncoated ELISA Kit | Affymetrix eBioscience | 88-7346-88 |
| Cell apoptosis detection ELISA^PLUS^ | MERCK | 11774425001 |
| Quant-iT™ PicoGreen™ dsDNA Quantitative Kit and dsDNA Reagent | Affymetrix eBioscience | P11496 |
| BCA Protein Assy Kit | Boytime | P0012 |
| Lymphoprep^TM^ Human lymphocyte isolate | Alere Technologies AS | 1114546 |
| Mouse bone marrow neutrophil isolation kit | Tbdscience | TBD2013NM |
| Pierce™ Cross linked magnetic IP/Co IP reagent kit | Invitrogen | 88805 |
| Anti-DYK0000 (Flag) immune magnetic beads | Selleck | B26101 |
| Pierce™ silver stain for Mass Spectrometry | Invitrogen | 24600 |
| Lipofectamine™ 3000 Transfection  Reagent | Invitrogen | L3000015 |
| FastPure EndoFree Plasmid Mini Kit | Vazyme | DC203-01 |
| Lysing Buffer | BD | 555899 |
| DMSO | Solarbio Science & Technology | D8370 |
| 1-Bromo-3-chloropropane | Sigma-Aldrich | B9673 |
| Fetal Bovine Serum | Gibco | 10091148 |
| RPMI 1640 medium | Gibco | C11875500BT |
| DMEM medium | Gibco | C11995500BT |
| Brefeldin A Solution | Biolegend | 420601 |
| eBioscience™ Flow type membrane breaking buffer (10X) | Invitrogen | 00-8333-56 |
| eBioscience™ Flow cytometry intracellular fixation buffer | Invitrogen | 00-8222-49 |
| 4′,6-diamidino-2-phenylindole, dihydrochloride (DAPI) | Solarbio Science & Technology | G1012 |

**Supplemental Table 4. Antibody tables.**

| **REAGENT or RESOURCE** | **SOURCE** | **IDENTIFIER** |
| --- | --- | --- |
| Anti-Myeloperoxidase (MPO) (EPR20257) | Abcam | ab208670 |
| Anti-Histone H3 (citrulline R2 + R8 + R17) (Cith3) | Abcam | ab5103 |
| Anti-Myeloperoxidase (2D4) | Abcam | ab90812 |
| Myeloperoxidase antibody/4A4 | Bio-RAD | 0400-0002 |
| c-Raf (D4B3J) Rabbit mAb | Cell Signaling Technology | 53745 |
| Anti-RAF1 (phospho 621) (EPR1521) | Abcam | ab157201 |
| Anti-RAF1 (phospho 259) | Cell Signaling Technology | 9421 |
| Anti-RAF1 (phospho 301) | Abcam | ab30570 |
| Src (36D10) Rabbit mAb | Cell Signaling Technology | 2109 |
| Recombinant Anti-SRC Family (photo Y418) antibody (EP503Y) | Abcam | ab40660 |
| Src pY418 Antibody, anti-human, REAfinity™ | Miltenyi Biotec | 130-106-680 |
| DYKDDDDK tag Polyclonal antibody (Binds to FLAG® tag epitope) | Proteintech | 20543-1-AP |
| MEK1/2 (L38C12) Mouse mAb | Cell Signaling Technology | 4694 |
| Phospho-MEK1/2 (Ser217/221) (41G9) Rabbit mAb | Cell Signaling Technology | 9154 |
| p44/42 MAPK (Erk1/2) (137F5) Rabbit mAb | Cell Signaling Technology | 4695 |
| Phospho-p44/42 MAPK (Erk1/2) (Thr202/Tyr204) (D13.14.4E) XP® Rabbit mAb | Cell Signaling Technology | 4370 |
| β-Actin (13E5) Rabbit mAb | Cell Signaling Technology | 4970 |
| Anti-rabbit IgG, HRP-linked Antibody | Cell Signaling Technology | 7074 |
| Rabbit (DA1E) mAb IgG XP® Isotype Control (Sepharose® Bead Conjugate) | Cell Signaling Technology | 3423 |
| PE/Cyanine7 anti-mouse CD45.2 Antibody | Biolegend | 109830 |
| APC anti-mouse/human CD11b Antibody | Biolegend | 101212 |
| APC/Cyanine7 anti-mouse Ly-6G Antibody | Biolegend | 127624 |
| PE/Cyanine7 anti-human CD45 Antibody | Biolegend | 368532 |
| PE/Cyanine5 anti-human CD16 Antibody | Biolegend | 302010 |
| Pacific Blue™ anti-human CD66b Antibody | Biolegend | 305112 |
| Src pY418 Antibody, anti-human, PE,REAfinity™ | Miltenyi Biotec | 130-106-680 |

**Supplemental Table 5. Primers were used for Q-PCR.**

| Primer name | Sequence |
| --- | --- |
| RAF1-F | TGGACTCAAAGATGCGGTGTT |
| RAF1-R | AAAACCCGGATAGTATTGCTTGT |
| ERK-F | GGTTGTTCCCAAATGCTGACT |
| ERK-R | CAACTTCAATCCTCTTGTGAGGG |
| 18S-F | GGAAGTGCACCACCAGGAGT |
| 18S-R | TGCAGCCCCGGACATCTAAG |
